# The SR-BI Partner PDZK1 Facilitates Hepatitis C Virus Entry

**DOI:** 10.1371/journal.ppat.1001130

**Published:** 2010-10-07

**Authors:** Nicholas S. Eyre, Heidi E. Drummer, Michael R. Beard

**Affiliations:** 1 Centre for Cancer Biology, SA Pathology, Adelaide, South Australia, Australia; 2 School of Molecular and Biomedical Science, University of Adelaide, Adelaide, South Australia, Australia; 3 Burnet Institute, Melbourne, Victoria, Australia; 4 Department of Microbiology and Immunology, University of Melbourne, Parkville, Victoria, Australia; 5 Department of Microbiology, Monash University, Clayton, Victoria, Australia; The Rockefeller University, United States of America

## Abstract

Entry of hepatitis C virus (HCV) into hepatocytes is a multi-step process that involves a number of different host cell factors. Following initial engagement with glycosaminoglycans and the low-density lipoprotein receptor, it is thought that HCV entry proceeds via interactions with the tetraspanin CD81, scavenger receptor class B type I (SR-BI), and the tight-junction proteins claudin-1 (CLDN1) and occludin (OCLN), culminating in clathrin-dependent endocytosis of HCV particles and their pH-dependent fusion with endosomal membranes. Physiologically, SR-BI is the major receptor for high-density lipoproteins (HDL) in the liver, where its expression is primarily controlled at the post-transcriptional level by its interaction with the scaffold protein PDZK1. However, the importance of interaction with PDZK1 to the involvement of SR-BI in HCV entry is unclear. Here we demonstrate that stable shRNA-knockdown of PDZK1 expression in human hepatoma cells significantly reduces their susceptibility to HCV infection, and that this effect can be reversed by overexpression of full length PDZK1 but not the first PDZ domain of PDZK1 alone. Furthermore, we found that overexpression of a green fluorescent protein chimera of the cytoplasmic carboxy-terminus of SR-BI (amino acids 479–509) in Huh-7 cells resulted in its interaction with PDZK1 and a reduced susceptibility to HCV infection. In contrast a similar chimera lacking the final amino acid of SR-BI (amino acids 479–508) failed to interact with PDZK1 and did not inhibit HCV infection. Taken together these results indicate an indirect involvement of PDZK1 in HCV entry via its ability to interact with SR-BI and enhance its activity as an HCV entry factor.

## Author Summary

Hepatitis C virus (HCV) infection is a major cause of serious liver disease, with approximately 170 million people infected worldwide. Although significant advances have been made in the characterization and development of novel therapeutics to combat HCV infection, there is still a great need for an improved understanding of the HCV lifecycle and potential future targets of antiviral therapy. HCV entry into hepatocytes involves numerous plasma membrane proteins including CD81, scavenger receptor class B type I (SR-BI), claudin-1 and occludin. Although these proteins may comprise the complete set of essential HCV entry factors, the secondary factors that influence the co-ordinated involvement of these proteins in HCV entry remain to be determined. Here we identify the SR-BI partner protein PDZK1 as an indirect regulator of HCV entry. Our results indicate that binding of PDZK1 to the cytoplasmic carboxy-terminus of SR-BI influences the receptor's involvement in HCV entry such that disruption of this interaction may represent a future target of therapeutic intervention.

## Introduction

It is estimated that approximately 170 million people worldwide are infected with hepatitis C virus (HCV); a major cause of serious liver disease. At present there is no preventative vaccine available and the widely preferred treatment regime of pegylated interferon alpha (IFN-α) and ribavirin in combination is expensive, causes adverse side effects and is only effective for a fraction of individuals. Despite significant advances in identification of novel antiviral agents that inhibit HCV replication and polyprotein processing, concerns remain regarding the toxicity of these compounds and the likelihood of development of antiviral resistance [1]. The rapidly increasing understanding of the HCV entry process and significant advances in the development and application of HIV entry inhibitors (for review see [2]) have lead to a growing appreciation that HCV entry is another promising target for future antiviral therapies.

The recent development of the retroviral HCV pseudoparticle system (HCVpp), in which HCV E1E2 glycoproteins are assembled onto retroviral cores [3], [4], [5], and the infectious HCV cell culture (HCVcc) system, in which the full viral lifecycle is recapitulated in cell culture [6], [7], [8], have allowed in-depth analysis of the HCV entry process. At present there is strong evidence to suggest that the essential HCV entry factors include the tetraspanin CD81 [5], [9], [10], [11], the class B scavenger receptor SR-BI [9], [12], [13], [14], and the tight-junction proteins claudin-1 and occludin [15], [16], [17], [18], [19], [20]. Considering that these proteins may comprise the complete set of essential HCV entry factors [18], it still remains to be determined what the relative involvement of each of these entry factors is and, beyond expression, what secondary factors influence the contribution of these proteins to HCV entry.

SR-BI is the major receptor for high-density lipoproteins (HDL) and mediates both bi-directional flux of free cholesterol between cells and lipoproteins and selective uptake of cholesteryl esters into cells from HDL (reviewed in [21]). The latter function is of greatest significance in the liver and steroidogenic tissues [22], [23], where SR-BI is most highly expressed [24]. Studies using rodents have revealed that hepatic SR-BI expression is subject to little transcriptional regulation but instead is largely regulated at the post-transcriptional level by its interaction with the cytoplasmic adaptor molecule PDZK1 (reviewed in [25]).

PDZK1, which is also known as NHERF3, CAP70, CLAMP and NaPi-Cap1, is a four PDZ domain-containing adaptor protein that is predominantly expressed in the liver, kidney and small intestines [26]. Since the demonstration that the extreme C-terminus of SR-BI interacts with the first N-terminal PDZ domain of PDZK1 [26], [27], a number of *in vivo* and *in vitro* studies have demonstrated the importance of this interaction to the plasma membrane content of SR-BI and its activity as an HDL receptor in hepatocytes. Strikingly, in PDZK1 knockout (KO) mice, hepatic levels of SR-BI protein are reduced by greater than 95% and plasma HDL-cholesterol is elevated [28] to a level that approaches those levels seen in SR-BI KO mice [29]. Although these effects suggested the involvement of PDZK1 in regulating the stability of SR-BI and its effective targeting to the plasma membrane in hepatocytes, further studies have since revealed that hepatic overexpression of SR-BI in SR-BI/PDZK1 double knockout mice results in effective targeting of SR-BI to the hepatocyte plasma membrane and restoration of apparently normal lipoprotein metabolism [30]. The authors of this work therefore concluded that PDZK1 is not essential for the correct localization and function of SR-BI in the liver but instead is required for maintenance of ‘steady state levels’ of hepatic SR-BI protein [30].

Recently the importance of PDZK1/SR-BI interaction to the total hepatic abundance, plasma membrane content and activity in lipoprotein metabolism of SR-BI has been further dissected by studies involving the hepatic overexpression of C-terminally truncated PDZK1 mutants in transgenic mice [31], [32]. While overexpression of the first PDZ domain (PDZ1) of PDZK1 could not rescue normal SR-BI expression and activity in PDZK1 KO mice, overexpression of PDZ1 in wildtype mice resulted in cytoplasmic retention of endogenous SR-BI and a commensurate effect on lipoprotein metabolism [32]. Further studies revealed that transgenic expression of all four PDZ domains of PDZK1 is required to restore normal SR-BI protein abundance, plasma membrane content and activity in lipoprotein metabolism in PDZK1 KO mice [31]. Collectively these results indicate that interaction of the C-terminus of SR-BI with PDZK1, in itself, is not sufficient to enhance total SR-BI expression, plasma membrane localization and function. Instead other features of PDZK1 appear to be necessary for its impact on SR-BI expression and function. These may include phosphorylation of Ser-509 in the C-terminus of PDZK1, which is associated with increased SR-BI abundance in rat hepatoma cell culture [33] and/or association with other proteins in a macromoleular complex.

The importance of the cytoplasmic carboxy-terminus of SR-BI to its involvement in HCV entry has been examined in two recent studies [12], [13]. Firstly, Grove *et al* reported that soluble E2 binding and HCVcc infection levels are enhanced by overexpression of SR-BI or SR-BII, a splice-variant of SR-BI which features an alternative cytoplasmic carboxy terminus [13]. However, overexpression of SR-BI was associated with increases in HCVcc infection levels that were several-fold higher than those observed for overexpression of SR-BII [13], suggesting that features of the cytoplasmic tail dictate the molecule's efficient involvement in HCV entry. Secondly, it has recently been reported that complementation of SR-BI expression in rodent hepatoma cells co-expressing human CD81 and claudin-1 rendered these cells susceptible to infection with HCVpp and that this effect was limited when SR-BI constructs bearing various mutations in the cytoplasmic carboxy-terminus were substituted [12]. In that study, however, the extreme carboxy-terminus of SR-BI and expression of PDZK1 were not found to be significant determinants of HCV entry levels.

In the present study we have examined the association of human SR-BI and PDZK1 and how this impacts upon HCV entry and replication. Specifically we show that the association between SR-BI and PDZK1 is important for efficient entry of HCV into hepatoma cells and suggest that disruption of this interaction may be a future target of anti-HCV therapy.

## Results

### Domains and residues involved in PDZK1/SR-BI association and subcellular localization

**Figure 1:**
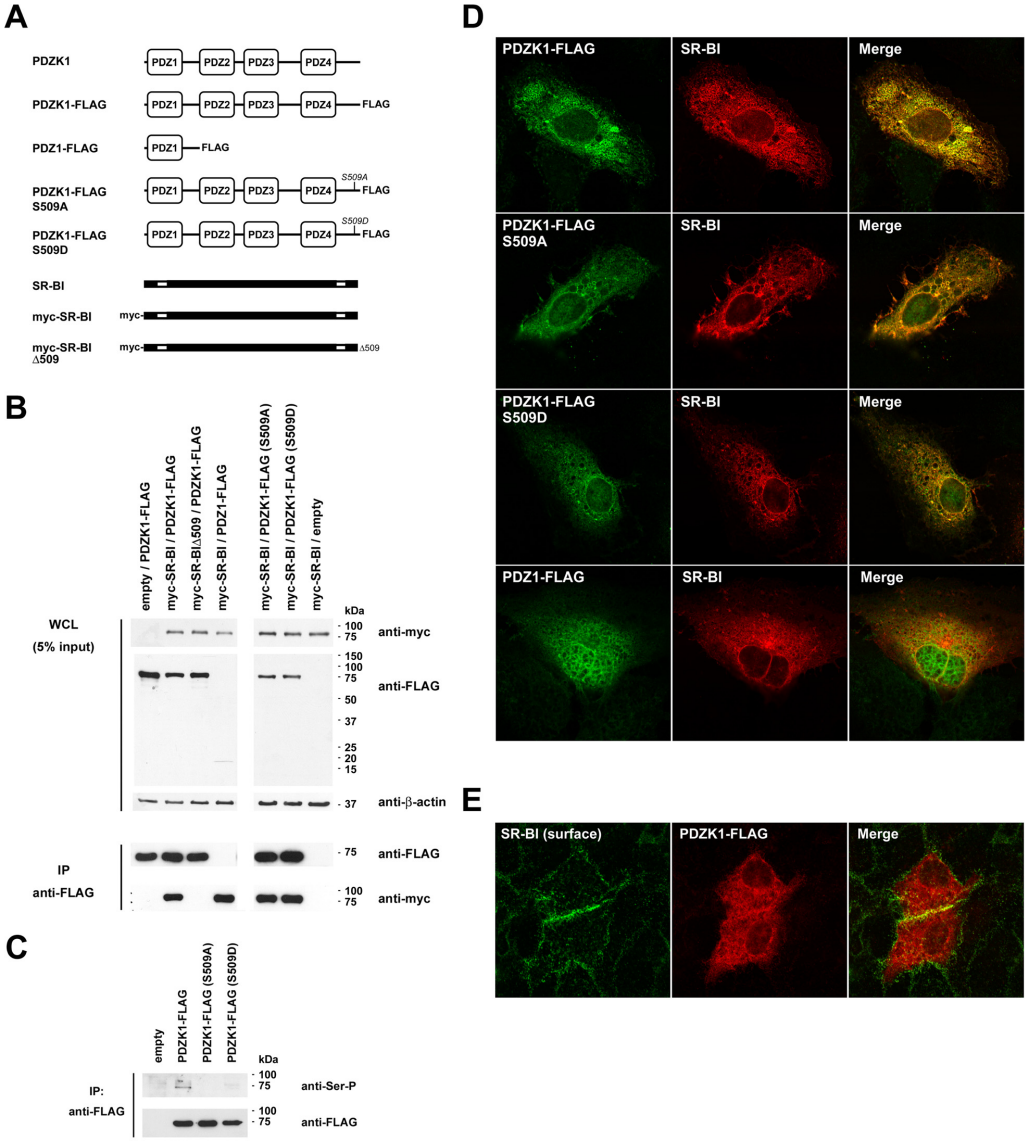
Interaction and colocalization of wildtype and mutant variants of SR-BI and PDZK1. (A) Schematic diagrams of PDZK1, SR-BI and epitope-tagged wildtype and mutant derivatives of each. For SR-BI schematic diagrams transmembrane regions that flank the large extracellular loop are depicted as white boxes. (B) Domains involved in SR-BI/PDZK1 interaction. 293T cells were co-transfected with the indicated SR-BI and PDZK1 expression constructs prior to immunoprecipitation with anti-FLAG antibody and Western analysis of immunoprecipitates using anti-FLAG or anti-Myc antibodies as indicated (lower panels). Note in immunoprecipitate samples PDZ1-FLAG (∼18 kDa) was not distinguishable from light chain bands (not shown). Expression of each of the FLAG- and Myc-tagged proteins, relative to the loading control β-actin, in whole cell lysates (WCL) prior to IP is shown in the upper panel. (C) Detection of serine phosphorylation of PDZK1. 293T cells were transfected with the indicated PDZK1 expression construct prior to immunoprecipitation (IP) with anti-FLAG antibody and Western analysis using anti-FLAG or anti-phosphoserine (SerP) antibodies as indicated. (D) Laser-scanning confocal microscopy (LSCM) analysis of colocalization of overexpressed PDZK1 constructs and full-length SR-BI. Huh-7 cells were grown on gelatin-coated coverslips, transfected with the indicated expression constructs and processed for indirect immunofluorescent detection of FLAG-tagged PDZK1 proteins (green) and SR-BI (red). Merged images (right panels) revealed colocalization (yellow). (E) LSCM analysis of the localization of surface endogenous SR-BI (green) and overexpressed wildtype PDZK1-FLAG (red). Merged images (right panel) revealed colocalization (yellow). For all conditions parallel samples were labelled with secondary antibody alone (surface SR-BI labelling) or isotype-matched irrelevant control antibodies to confirm specificity of labelling (not shown).

To date studies of the interaction of SR-BI and PDZK1 and the functional significance of this interaction have been performed using rodents and rodent-derived cell lines. To confirm the interaction of human SR-BI and PDZK1 and to examine the requirement of certain domains of each protein for the interaction, co-immunoprecipitation (co-IP) experiments were performed. For this, 293T cells were co-transfected with expression plasmids encoding wildtype or mutant variants of SR-BI and PDZK1 (Figure 1A) prior to immunoprecipitation of FLAG-tagged PDZK1 proteins and immunoblot detection of Myc-tagged SR-BI proteins (Figure 1B; lower panel). In these experiments 293T cells were used as they can be transiently transfected at high efficiency, therefore allowing ready detection of overexpressed proteins in whole cell lysates and immunoprecipitates. As expected, wildtype SR-BI readily co-immunoprecipitated with wildtype PDZK1, while a truncated SR-BI variant that lacks the final carboxy-terminal lysine residue (mycSR-BIΔ509) was not detectable in immunoprecipitates of co-transfected wildtype PDZK1. Furthermore we confirmed that the first amino-terminal PDZ domain of PDZK1 (PDZ1) was sufficient to co-IP wildtype SR-BI, with the NYGF motif present at amino acid residues 19–22 of PDZ1 being a predicted site of interaction with the carboxyl terminus of SR-BI. Next we examined whether phosphorylation of PDZK1 at Ser-505 impacted upon PDZK1/SR-BI association. For the sake of consistency with the previous report of phosphorylation of the corresponding site in rodent PDZK1 [33], this site will henceworth be referred to as Ser-509. While mutation of this site (S509A and S509D) resulted loss of serine phosphorylation in 293T cells (Figure 1C), both phosphodefective (S509A) and phosphomimetic (S509D) mutants of PDZK1 associated with wildtype SR-BI with no apparent difference in the relative amount of SR-BI that was co-immunoprecipitated with these PDZK1 mutants (Figure 1B). Next, given that the C-terminus of SR-BI has been implicated in its dimerization and multimerization [34], we investigated whether the PDZK1-interacting domain at the C-terminus of SR-BI was involved in receptor dimerization. Co-IP studies in transfected 293T cells revealed that mycSR-BIΔ509 readily interacts with FLAG-tagged full-length SR-BI and FLAG-SR-BIΔ509, suggesting that PDZK1-interaction is not involved in homodimerization of SR-BI (Figure S1, A). Similarly, co-IP studies in Huh-7 cells confirmed dimerization of FLAG-SR-BI with mycSR-BIΔ509 and the ability of mycSR-BI to interact with PDZK1-FLAG in this cell type (Figure S1, B). Accordingly confocal analysis of the localization of full length SR-BI and SR-BIΔ509 revealed no appreciable difference in the localization of these proteins in transfected Huh-7 cells (Figure S1, C).

Finally, confocal analysis revealed extensive co-localization of non-tagged wildtype SR-BI with FLAG-tagged PDZK1 in the cytoplasm of transfected Huh-7 hepatoma cells (Figure 1D), with no apparent effect of mutation of PDZK1 on its localization or co-localization with SR-BI for each of the mutants S509A and S509D. However, overexpressed PDZ1 displayed additional nuclear localization, despite substantial co-localization with cytoplasmic SR-BI. To reveal the localization of surface-displayed endogenous SR-BI with respect to overexpressed PDZK1-FLAG, transfected Huh-7 cells were fixed with formalin and SR-BI was labelled by indirect immunofluorescence with an antibody that recognizes the extracellular loop of SR-BI (and SR-BII) prior to subsequent fixation, permeabilization and labelling of PDZK1-FLAG (Figure 1E). These studies revealed a small degree of overlap in the localization of surface SR-BI and PDZK1-FLAG that was most evident at sites of cell-cell contact. Taken together these experiments demonstrate that the interaction of SR-BI with PDZK1 is not dependent on Ser-509 phosphorylation of PDZK1 and that the disruption of this interaction by truncation of SR-BI cannot be attributed to an appreciable alteration in its localization.

### Stable knockdown of PDZK1 expression in Huh-7 hepatoma cells inhibits HCV entry

**Figure 2:**
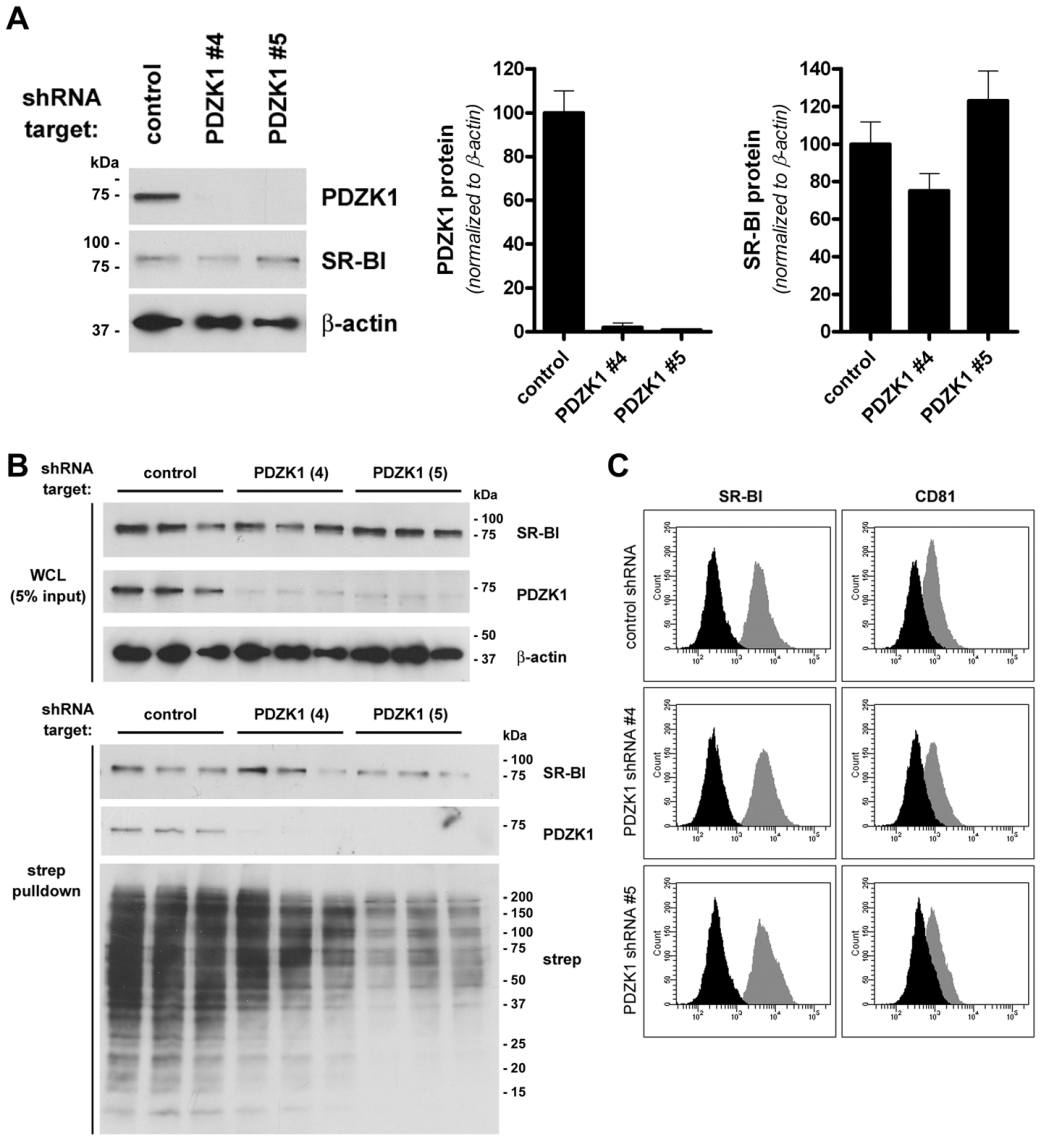
Stable knockdown of PDZK1 expression in Huh-7 cells. (A) Huh-7 cells were stably transduced with lentiviral shRNA expression vectors encoding a non-target shRNA control or shRNA's specific for PDZK1 (4 and 5), before Western analysis of expression of PDZK1 (∼70 kDa) and SR-BI (∼85 kDa). β-actin (∼42 kDa) was used as a loading control. PDZK1 and SR-BI protein levels were quantified by densitometry and normalized to those of the loading control β-actin (graphs). Data are means + SEM (n = 3). (B) Cell-surface expression levels of SR-BI. Huh-7 cells expressing the indicated shRNA targets were surface-biotinylated prior to detergent lysis, streptavidin-precipitation of biotinylated proteins and Western blotting. SR-BI and PDZK1 protein levels in whole cell lysates (WCL) (upper panels) and streptavidin-precipitates (lower panels) are shown. (C) Flow cytometric analysis of surface levels of SR-BI/II (left panel gray histograms) and CD81 (right panel gray histograms) in Huh-7 shRNA cell lines. Background fluorescence levels (black histograms) were determined by labelling cells with secondary antibody only (SR-BI staining) or an irrelevant isotype-matched control antibody (CD81 staining). Results are representative of two separate experiments performed in triplicate.

Having confirmed that human SR-BI and PDZK1 interact we next investigated the involvement of PDZK1 in entry of HCV using stable shRNA-knockdown of endogenous expression of PDZK1 in Huh-7 cells. Despite effective knockdown of PDZK1 expression, total SR-BI protein levels were not significantly altered (Figure 2A). Furthermore, cell-surface biotinylation experiments demonstrated that SR-BI remained readily detectable in streptavidin precipitates of plasma membrane proteins in PDZK1-knockdown cell lines (Figure 2B). Interestingly PDZK1 was co-precipitated with biotinylated plasma membrane proteins purified from control shRNA-expressing cells, but not from PDZK1 knockdown cells indicating that residual endogenous PDZK1 in the latter cells is not preferentially plasma membrane-associated (Figure 2B). Since PDZK1 has no transmembrane domains it is likely that enrichment of PDZK1 in streptavidin precipitates in these studies is the result of its association with the cytoplasmic tail of SR-BI at the inner leaflet of the plasma membrane and possibly other transmembrane partner proteins present in these cells. Furthermore, flow cytometric analysis of surface levels of SR-BI and splice variant SR-BII revealed no impact of PDZK1 knockdown on surface levels of these proteins (Figure 2C). Likewise surface levels of CD81 were not significantly altered by PDZK1 knockdown (Figure 2C). Interestingly, confocal analysis of surface-labelled cells revealed extensive colocalization of SR-BI and CD81 which was not appreciably altered by PDZK1 knockdown (Figure S2, A). Similarly we did not note any changes in the staining pattern of occludin in PDZK1 knockdown cells compared to control cells (Figure S2, B). Given that the extracellular domain of SR-BI is shared by splice-variant SR-BII [35], experiments involving confocal analysis of the localization of intracellular SR-BI and surface SR-BI/II in the same samples were undertaken to confirm that the majority of surface SR-BI/II staining is also co-stained with an antibody directed against the SR-BI-specific cytoplasmic C-terminus (Figure S3). From these images it was also apparent that PDZK1 knockdown does not cause an appreciable shift in the proportion of intracellular and surface SR-BI staining. Together these results indicate that near-complete loss of PDZK1 expression in Huh-7 cells does not significantly alter total or surface levels of SR-BI or its localization with respect to other requisite entry factors CD81 and occludin at the cell surface.

**Figure 3:**
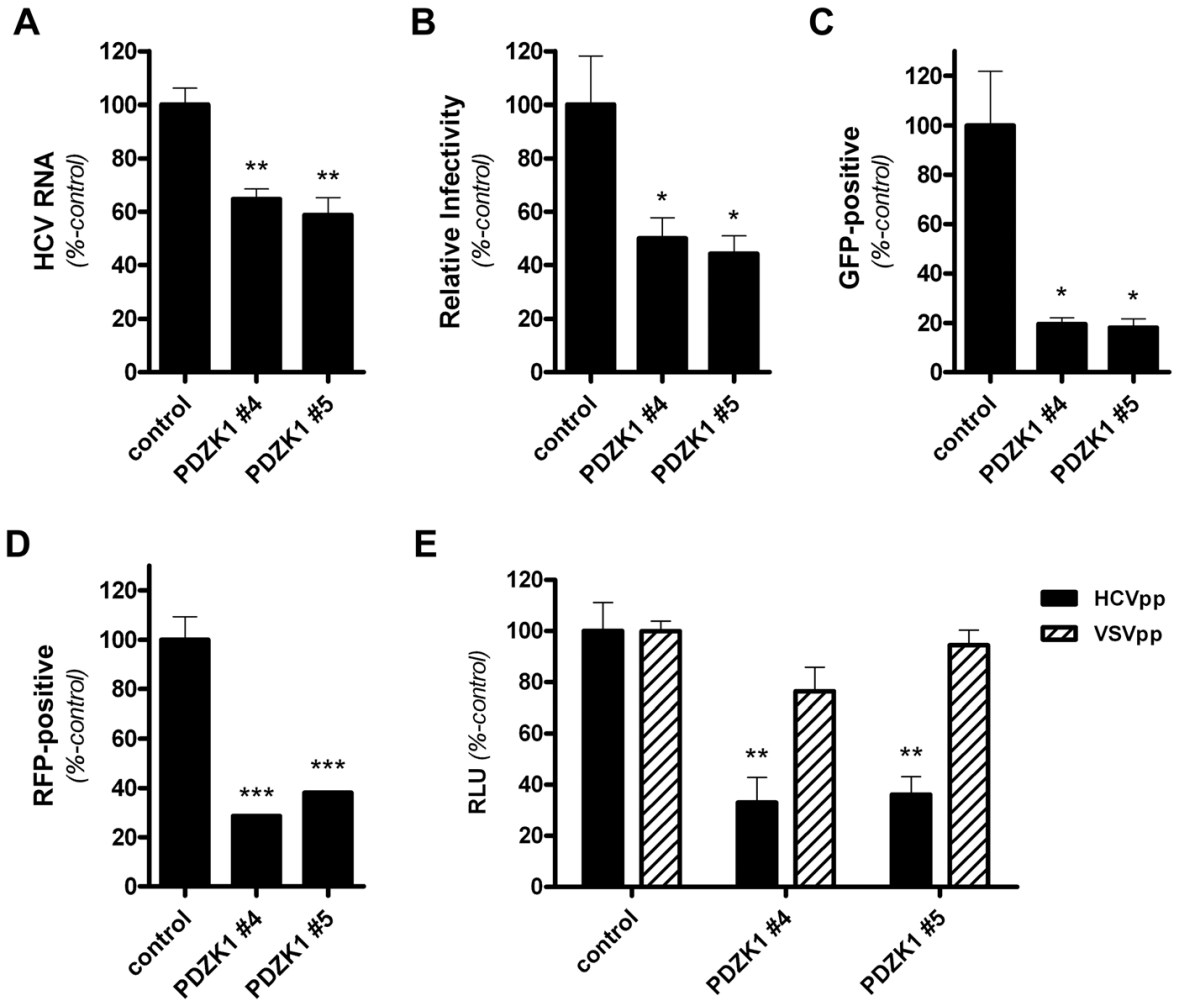
HCV entry is inhibited by stable knockdown of PDZK1 expression in Huh-7 cells. (A) Huh-7 cells stably expressing a non-target control shRNA or PDZK1-specific shRNA constructs (#4 and #5) were incubated with HCVcc (JFH1; approximate multiplicity of infection: 0.1) for 3 h prior to washing and return to culture for 72 h. HCV RNA levels, normalized to those of the cellular housekeeping gene RPLPO, were quantified by real-time RT-PCR and expressed as a percentage of control HCV RNA levels. (B) Huh-7 cells expressing the indicated shRNA constructs were incubated with HCVcc (JFH1; approximate multiplicity of infection: 0.1) for 3 h, washed and returned to culture for 72 h prior to enumeration of NS5A-positive foci of infected cells. Virus infectivity is expressed relative to those of cells expressing the non-target shRNA control. (C and D) Huh-7 shRNA cell lines were incubated with HCVcc (Jc1/GFP [C] of Jc1/RFP [D]) for 72 h prior to quantification of NS5A-associated epifluorescence (GFP or RFP) by flow cytometry. HCVcc infection levels are expressed relative to those of cells expressing the non-target shRNA control. (E) Huh-7 cells expressing the indicated shRNA constructs were challenged with HCVpp and VSVGpp encoding Luciferase reporters, washed and returned to culture for 72 h prior to quantification of luciferase activity. Specific HCVpp and VSVpp infectivity levels were calculated by subtraction of the signals associated with non-enveloped pseudoparticles (Env-pp). Values are expressed relative to infectivity levels for cells expressing the control non-target shRNA. For each graph data are means + SEM (n = 4). *, *P*<0.05; **, *P*<0.01; ***, *P*<0.001.

Next we investigated the influence of stable knockdown of PDZK1 expression in Huh-7 cells on HCV entry and replication. Following infection of these cells with cell culture propagated HCV (HCVcc; JFH-1), HCV RNA levels were approximately 40% lower in the PDZK1-knockdown cell lines compared to cells that expressed a non-targeting shRNA control (Figure 3A). Likewise, the relative susceptibility of these cells to HCVcc infection was approximately 50% lower than control cells, as determined by enumeration of HCV-positive foci (focus forming units/ml; FFU/ml) following indirect immunofluorescent labelling (Figure 3B). Similarly infection of these cells with Jc1/GFP or Jc1/RFP infectious HCV chimeras, that bear the GFP- or RFP-coding sequences inserted in-frame into domain III of NS5A of the infectious genotype 2a chimera Jc1 [36], and flow cytometric analysis of GFP- or RFP-associated epifluorescence revealed that PDZK1-knockdown cells were approximately 80% (Jc1/GFP) and 60% (Jc1/RFP) less susceptible to infection with these chimeras than their control counterparts (Figures 3C and 3D). To confirm that the observed effects were attributable to an impact of PDZK1-knockdown on HCV entry, and not an effect on HCV replication or spread, HCVpp (H77s) entry into these cells was measured. As for HCVcc infections, HCVpp entry was significantly reduced by PDZK1 knockdown (Figure 3E). In these experiments VSVG-pseudoparticle infection levels were unchanged by PDZK1 knockdown, indicating that PDZK1 knockdown causes HCV-specific inhibition of viral entry. Further evidence that HCV entry, and not HCV replication, is limited by PDZK1 knockdown was provided by the demonstration that stable knockdown of PDZK1 expression in Huh-7 cells that harbour the genome-length NNeo/C-5B(RG) replicon did not significantly impact upon HCV RNA levels or HCV-associated immunofluorescence (Figure S4). From these experiments we conclude that expression of PDZK1 contributes to HCV entry into Huh-7 cells most probably via an influence on its binding partner SR-BI despite no dramatic effects on total or cell surface levels of SR-BI protein. Given our observations that PDZK1 knockdown is associated with decreased HCV entry levels for both genotype 1a (HCVpp) and 2a (HCVcc) infection systems and the high degree of divergence between E1/E2 sequences for these genotypes, it is likely that the observed involvement of PDZK1 will hold true for all HCV genotypes. However, it has been reported that the relative involvement of SR-BI in HCV entry can differ between HCV genotypes and subtypes [37] and thus the relative influence of PDZK1 on SR-BI-dependent HCV entry may differ between HCV genotypes/subtypes accordingly.

### The impact of PDZK1 knockdown on HCV entry in polarized HepG2 hepatoma cells

**Figure 4:**
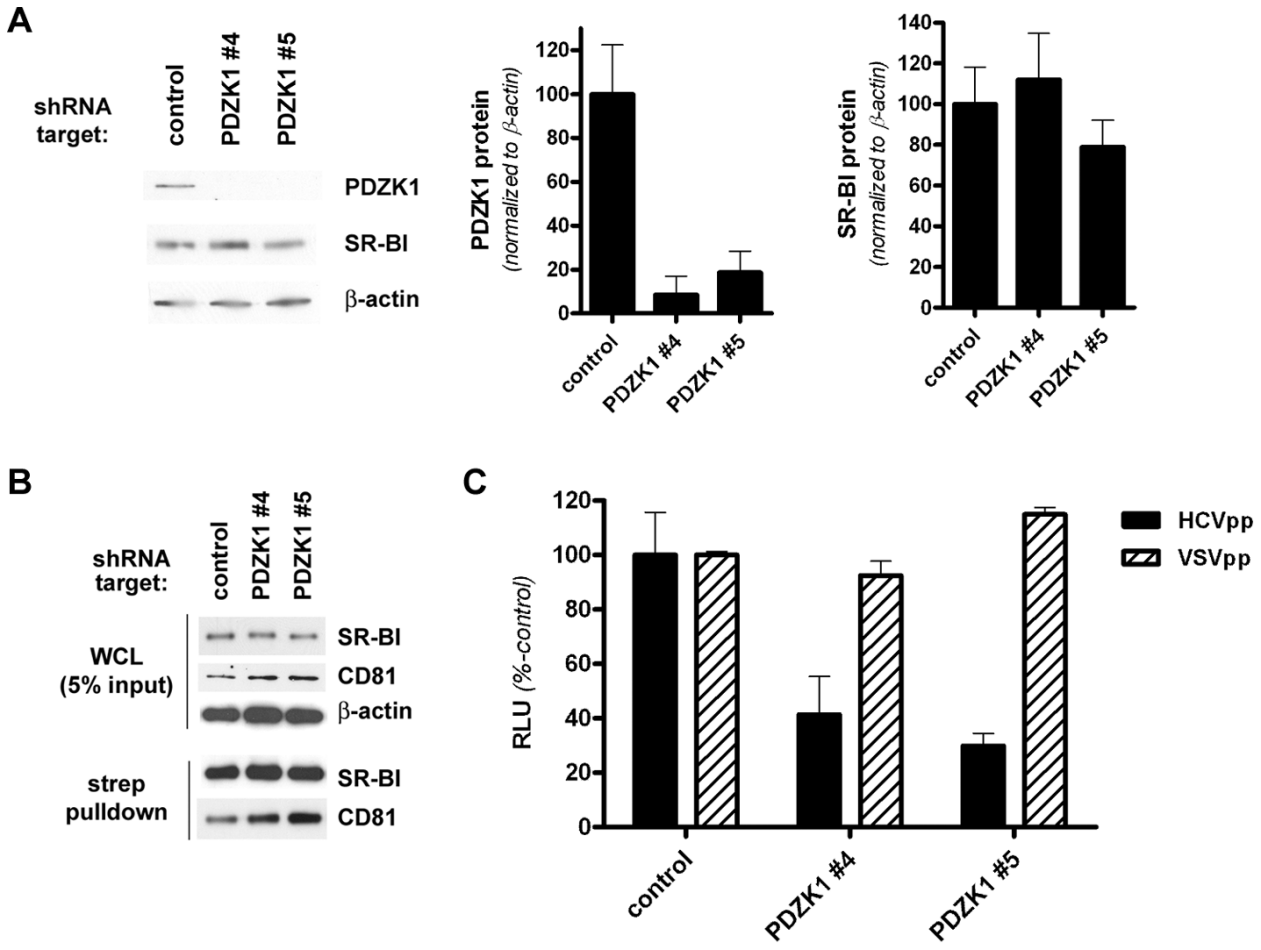
Stable knockdown of PDZK1 expression in HepG2(N6)+CD81 cells. (A) HepG2(N6)+CD81 cells expressing the indicated shRNA constructs were cultured under polarizing conditions (5 d post-confluence) prior to Western analysis of PDZK1 (∼70 kDa) and SR-BI (∼85 kDa) protein levels. β-actin (∼42 kDa) was used as a loading control. PDZK1 and SR-BI protein levels were quantified by densitometry and normalized to those of the loading control β-actin (graphs). Data are means + SEM (n = 3). (B) Cell-surface expression levels of SR-BI. HepG2(N6)+CD81 cells (5 d post-confluence) expressing the indicated shRNA targets were surface-biotinylated prior to detergent lysis, streptavidin-precipitation of biotinylated proteins and Western blotting. SR-BI, PDZK1 and CD81 (∼26 kDa) protein levels in whole cell lysates (WCL) (upper panels) and streptavidin-precipitates (lower panels) are shown. Overexpressed CD81 (with a C-terminal Myc epitope tag) was detected with an anti-C-myc antibody. (C) Confluent HepG2(N6)+CD81 cells expressing the indicated shRNA constructs were infected with HCVpp or VSVpp 72 h prior to measurement of luciferase activity. Data are means + SEM (n = 4).

Given the contrasting effects on total and cell surface SR-BI protein levels of PDZK1 knockdown in Huh-7 cells and PDZK1 gene knockout in mice [28] and the past demonstration that ectopically expressed SR-BIdel509 functions similarly to wildtype SR-BI in transfected cell lines but is unstable and mislocalized in transgenic mouse liver [27], we set about generating a polarized HepG2 cell culture model which, in some respects, would more accurately reflect the *in vivo* liver situation than non-polarized Huh-7 cells. To this end we stably overexpressed CD81 in the HepG2(N6) cell line, which displays simple columnar polarity [38], to render these cells permissive to HCVcc and HCVpp infection [9], [11], [39], [40]. Following confirmation of high-level cell surface expression of CD81 (not shown), these cells were stably transduced with PDZK1-specific or non-target control lentiviral shRNA vectors. As for Huh-7 cells, stable knockdown of endogenous PDZK1 expression in HepG2(N6)+CD81 cells that had been grown under polarizing conditions (5 days post-confluency) did not significantly alter total levels of SR-BI protein (Figure 4A). Likewise there was no discernable impact of PDZK1 knockdown on cell surface levels of SR-BI or CD81, as determined by Western analysis following cell-surface biotinylation and streptavidin precipitation of plasma membrane associated proteins (Figure 4B). However, we found that PDZK1 knockdown was associated with a moderate yet significant reduction in the susceptibility of HepG2(N6)+CD81 cells to HCVcc (Jc1/Myc) infection (not shown). Moreover HCVpp infection was substantially reduced in confluent HepG2(N6)+CD81 cells that expressed PDZK1-specific shRNA's compared to control shRNA-expressing counterparts (Figure 4C). Taken together these results demonstrate that knockdown of endogenous PDZK1 expression in HepG2(N6)+CD81 cells results in their reduced susceptibility to HCV infection.

### Overexpression of full-length PDZK1 restores efficient HCVcc entry in PDZK1-knockdown Huh-7 cells

**Figure 5:**
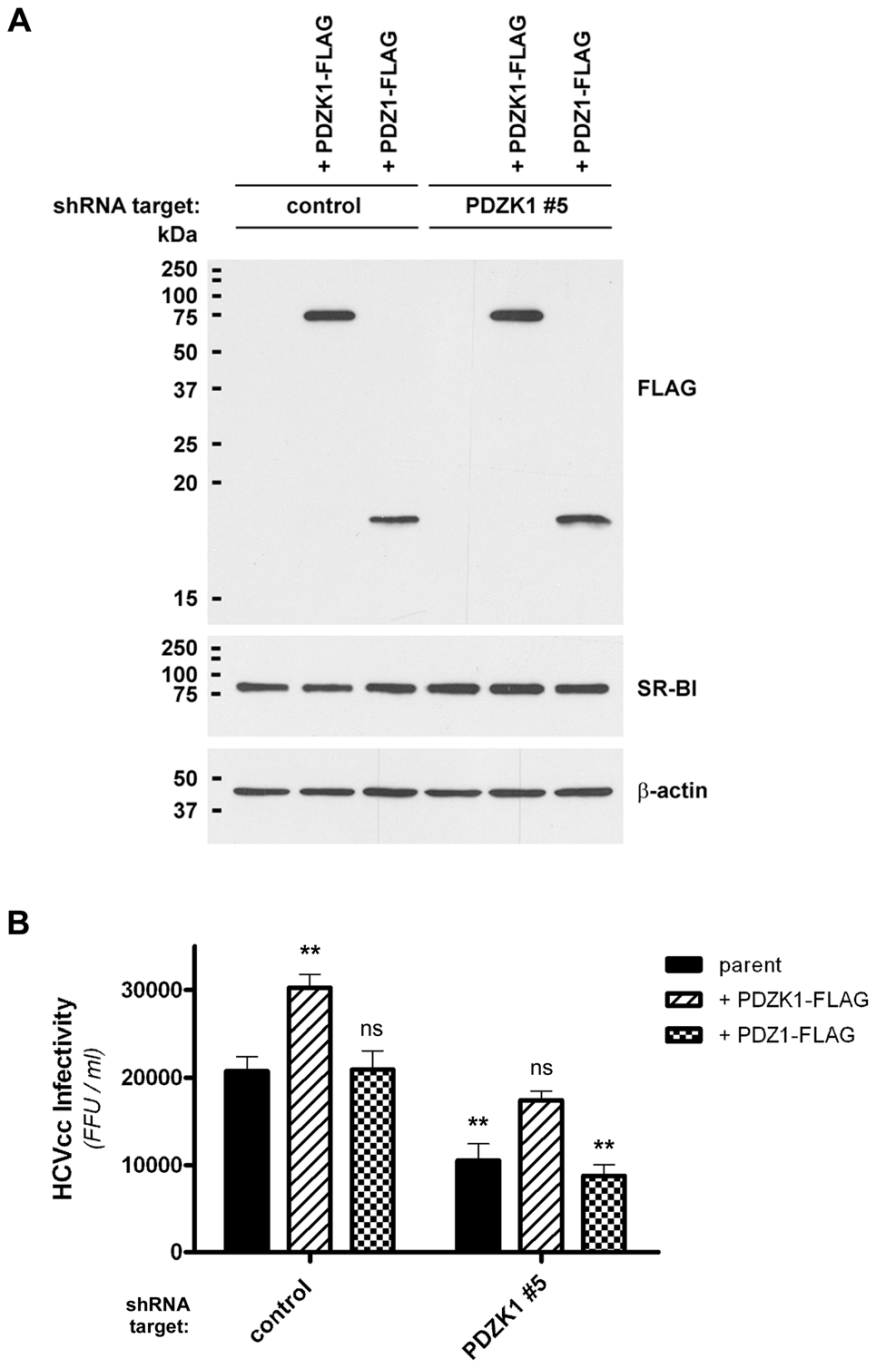
Expression of full-length PDZK1 restores normal HCVcc infection levels in PDZK1-knockdown Huh-7 cells. (A) Huh-7 cells expressing the indicated shRNA construct were stably transduced with shRNA-refractory PDZK1-FLAG or PDZ1-FLAG expression constructs prior to Western blot analysis of FLAG-tagged transgene and SR-BI protein levels. β-actin was used as a loading control. (B) Huh-7 cells stably expressing the indicated shRNA and cDNA constructs were incubated with HCVcc (Jc1/Myc; approximate multiplicity of infection: 0.3) for 3 h, washed and returned to culture for 72 h, prior to detection of NS5A-positive foci of infected cells by immunofluorescence using an anti-Myc antibody. Virus infectivity is expressed as the number of focus-forming units (FFU)/ml. Data are means + SEM (n = 4). Statistically significant differences from parent cells expressing the non-target shRNA control are indicated by asterisks (**, *P*<0.01; ns, not significant).

To further confirm the involvement of PDZK1 in HCV infection we generated a lentiviral PDZK1-FLAG expression construct bearing silent mutations (S^tca^261S^agc^) to render the encoded transcripts refractory to shRNA silencing by PDZK1 shRNA#5. This construct was then overexpressed in both non-target shRNA control and PDZK1 shRNA#5 Huh-7 cell lines, to examine whether overexpression of the shRNA-refractory PDZK1 construct would impact upon HCVcc infection and whether ‘normal’ levels of HCVcc infection could be restored. In these experiments a PDZ1-FLAG lentiviral expression construct served as an additional control that was expected to interact with the C-terminus of SR-BI but not restore PDZK1 function in PDZK1 knockdown cells. Following generation of stable cell lines using these vectors Western analysis showed strong expression of PDZK1-FLAG and PDZ1-FLAG that was comparable between each of the cell lines and did not alter total endogenous SR-BI protein levels (Figure 5A), while parallel immunofluorescent labelling of the overexpressed FLAG-tagged proteins indicated that over 70% of cells expressed these constructs for each of the cell lines (not shown).

Infection of these cells with HCVcc (Jc1/Myc) and quantification of HCV-positive foci three days later revealed that overexpression of full-length PDZK1 significantly increased HCVcc infection levels in shRNA control cell lines and restored levels of HCVcc infection in PDZK1 shRNA#5-expressing cells to levels approaching those of the parental non-target shRNA control cells (Figure 5B). In contrast to the effects of overexpression of full-length PDZK1-FLAG, overexpression of PDZ1-FLAG did not restore HCVcc infection levels in PDZK1 shRNA#5 cells to those of non-target shRNA control cells. Given that hepatic expression of a similar murine PDZ1 construct is reported to cause mislocalization of endogenous SR-BI in transgenic mice and a commensurate effect on HDL metabolism [32], we anticipated that overexpression of PDZ1-FLAG would have a similar dominant-negative effect on HCVcc infection. In contrast we did not observe any significant impact of PDZ1-FLAG expression on the susceptibility of either non-target shRNA- or PDZK1 shRNA-expressing cells to HCVcc infection. However, we have observed that the PDZ1 polypeptide used in this study appears somewhat instable compared to full-length PDZK1 (for example see Figure 1B) and it is possible that high-level overexpression of an alternative construct that encodes a more stable PDZ1 variant may cause the inhibitory effects on HCV entry that were predicted. Taken together these data indicate that the ability of PDZK1 to enhance HCV infection requires regions of the protein that lie outside the SR-BI-interacting domain of the molecule.

### The cytoplasmic carboxy-terminus of SR-BI is sufficient to interact with PDZK1 and influence the subcellular localization of a soluble reporter

**Figure 6:**
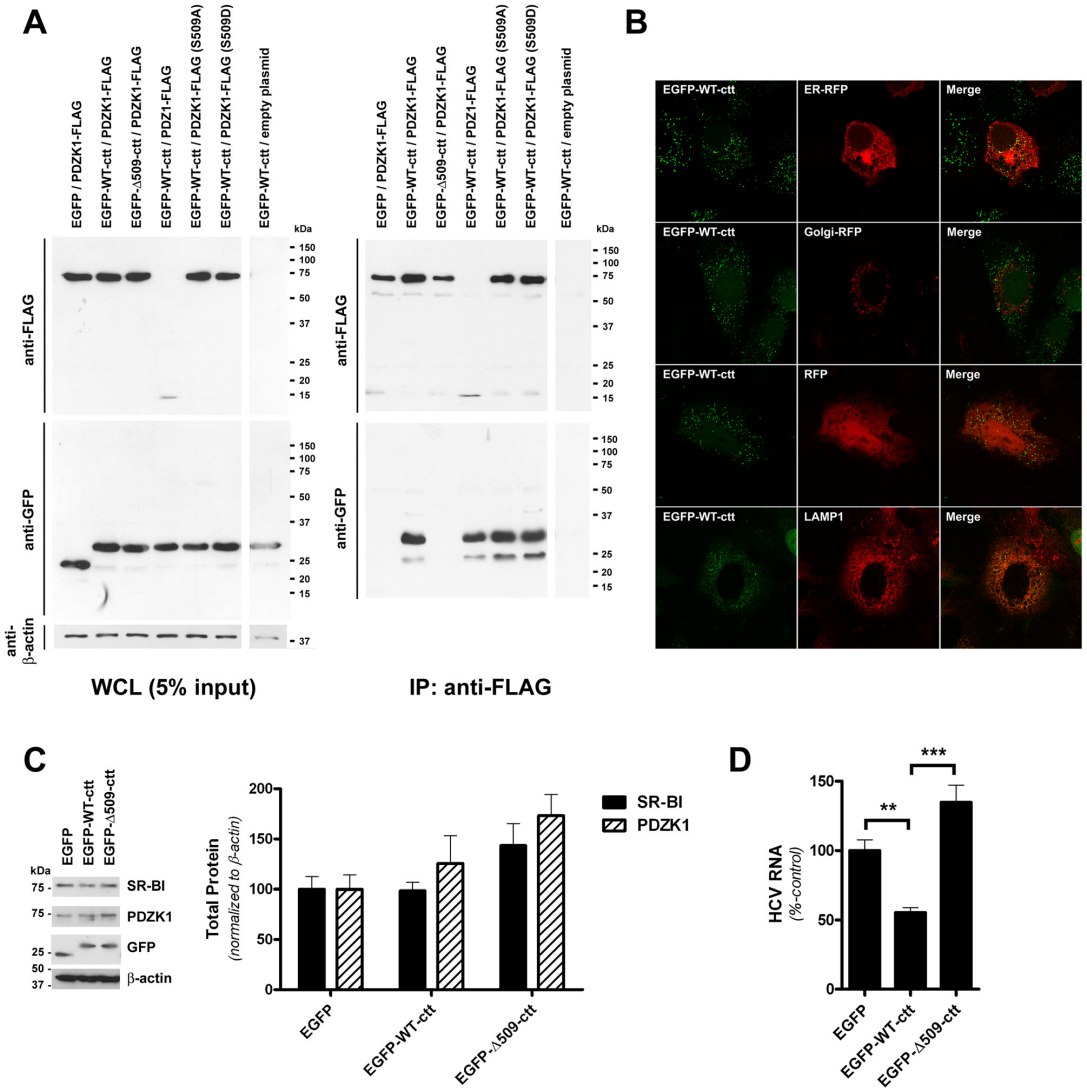
A green fluorescent protein chimera of the SR-BI C-terminus interacts with PDZK1 and inhibits HCVcc infection. (A) 293T cells were co-transfected with expression vectors encoding the indicated chimeric GFP-SR-BI cytoplasmic C-terminus (cct) (aa 479-509 or 479-508) expression construct and PDZK1 expression construct prior to immunoprecipitation with anti-FLAG antibody and Western analysis of immunoprecipitates using anti-FLAG or anti-GFP antibodies as indicated (right panels). Expression of each of the FLAG- and GFP-tagged proteins, relative to the loading control β-actin, in whole cell lysates (WCL) prior to IP is shown in the left panel. (B) Laser-scanning confocal microscopy analysis of the localization of EGFP-WT-ctt. Huh-7 cells stably expressing EGFP-WT-ctt were grown on gelatin-coated coverslips, transfected with the indicated red fluorescent protein (RFP) expression construct (where applicable) and processed for direct detection of fluorescent protein-associated epifluorescence or indirect immunofluorescent detection of LAMP1. Merged images are shown in the right panels. (C) Western analysis of SR-BI (∼85 kDa), PDZK1 (∼70 kDa), EGFP (∼25 kDa), EGFP-WT-ctt (∼32 kDa) and EGFP-Δ509-ctt (∼32 kDa) protein levels in Huh-7 cells stably expressing the indicated EGFP construct. β-actin (∼42 kDa) was used as a loading control. SR-BI and PDZK1 protein levels were quantified by densitometry and normalized to those of β-actin (graph inset). Data are means + SEM (n = 3). (D) Huh-7 cells stably expressing the indicated EGFP chimera were incubated with HCVcc (Jc1/Myc; approximate multiplicity of infection: 0.3) for 3 h, washed and returned to culture for 72 h prior to quantification of HCV RNA levels (normalized to RPLPO mRNA). HCV RNA levels are expressed relative to those of EGFP-expressing control cells. Data are means + SEM (n = 4). **, *P*<0.005; ***, *P*<0.001.

To further investigate the domains of each protein involved in SR-BI/PDZK1 association and to investigate the validity of a putative dominant-negative inhibitor of the interaction, the C-terminal 30 amino acids of the cytoplasmic carboxy-terminus of wildtype SR-BI (WT-ctt) were appended in-frame to the carboxy-terminus of enhanced green fluorescent protein (EGFP). As for full-length SR-BI, the EGFP-WT-ctt chimera was readily detectable in co-immunprecipitates of co-transfected wildtype PDZK1 (Figure 6A). Importantly, a control chimera that lacked the final C-terminal lysine residue (EGFP-Δ509-ctt) did not co-IP with wildtype PDZK1. As for full-length SR-BI, EGFP-WT-ctt was co-immunoprecipitated with wildtype PDZK1, PDZ1, PDZK1 S509A and PDZK1 S509D.

Next, Huh-7 cell lines that stably express EGFP, EGFP-WT-ctt or EGFP-Δ509-ctt were generated. Confocal analysis of the localization of these fluorescent protein chimeras revealed that EGFP-WT-ctt localized to distinct cytoplasmic punctae that partially co-localized with LAMP1, a resident protein of late endosomes and lysosomes (Figure 6B). In contrast, EGFP-Δ509-ctt, which does not interact with PDZK1, was indistinguishable in localization to unmodified EGFP (Figure S5, B), suggesting that the distinctive punctate cytoplasmic localization of EGFP-WT-ctt could be attributed to its ability to interact with endogenous PDZK1. Western analysis of these cells revealed that stable expression of EGFP-WT-ctt had no significant effect on the total endogenous PDZK1 and SR-BI content in Huh-7 cells compared to cells that stably expressed unmodified EGFP or EGFP-Δ509-ctt (Figure 6C). Similarly, cell surface levels of SR-BI were not appreciably altered by expression of EGFP-WT-ctt, compared to those of cell lines expressing EGFP or EGFP-Δ509-ctt (Figure S5, A). Nevertheless we reasoned that EGFP-WT-ctt would interact with endogenous PDZK1 and, at high expression levels, out-compete endogenous SR-BI for PDZK1 binding. In agreement with this theory Huh-7 cells that stably expressed EGFP-WT-ctt were nearly 50% less susceptible to HCVcc infection than native EGFP-expressing control counterparts and nearly 80% less susceptible to HCVcc infection than Huh-7 cells stably expressing EGFP-Δ509-ctt (Figure 6D). Altogether these results indicate that expression of the cytoplasmic carboxy terminus of SR-BI, as a fusion to a soluble reporter, causes an inhibition of HCVcc infection that can be attributed to its ability to bind PDZK1.

## Discussion

Recent studies have indicated that the intracellular domains of SR-BI, particularly the cytoplasmic C-terminus, contribute to the activity of the receptor in HCV entry [12], [13], indicating that the binding of HCV to the extracellular domain of SR-BI, in itself, is not sufficient for the efficient involvement of SR-BI in the HCV entry process. Instead the cytoplasmic regions of the molecule may dictate dynamic changes in receptor localization and/or interactions with other HCV entry factors that facilitate HCV entry. In the context of studies of the involvement of SR-BI in HDL metabolism in mice, tissue-specific interaction of the cytoplasmic C-terminus of SR-BI with the adaptor protein PDZK1 has emerged as an important determinant of the receptor's effective enrichment at the hepatocyte plasma membrane and activity in HDL-CE transport (reviewed in [25]).

In the present study we have examined the association of human variants of SR-BI and PDZK1 and the importance of this interaction to HCV entry. We have confirmed the interaction of SR-BI with PDZK1 and provide evidence that the extreme C-terminus of SR-BI and the first PDZ domain of PDZK1 are critical sites of the interaction. Accordingly these proteins were found to overlap extensively in subcellular localization in the cytoplasm of transfected Huh-7 cells, with no discernable impact of mutation of the sites of interaction in both proteins or the major site of phosphorylation of PDZK1 on the colocalization of these proteins. While it has been reported that the protein kinase A (PKA)-dependent phosphorylation of the corresponding serine residue in rat PDZK1 is necessary for the ability of overexpressed PDZK1 to increase endogenous SR-BI protein levels in Fao hepatoma cells [33], we found no discernable impact of phosphodefective or phosphomimetic mutations at this site on the interaction or localization of these proteins, suggesting that phosphorylation at this site causes more subtle effects on SR-BI biology in human hepatocytes. It will be interesting to examine the relative importance of phosphorylation of PDZK1 to the involvement of SR-BI in the HCV entry process.

Effective knockdown of PDZK1 expression in Huh-7 cells was associated with a decreased susceptibility of these cells to infection with HCVcc and HCVpp, despite no discernable impact on total or surface levels of SR-BI protein. These effects contrast the more dramatic effects of PDZK1-knockout on the SR-BI protein content in mouse liver [28] and suggest that the plasma membrane-associated SR-BI in these cells is impaired in its ability to facilitate HCV entry. It is possible that loss of interaction with PDZK1 alters the localization of SR-BI within the plasma membrane. For example, it has been reported that SR-BI localizes to caveolae, a specialized subset of lipid raft domains of the plasma membrane, and that this localization may be important to the receptor's activity [41]. Consistent with this theory, removal of membrane raft cholesterol with ΜβCD inhibits HCV entry [42]. However, it has also been reported that neither SR-BI, nor other requisite entry factors CD81 and claudin-1, are strongly associated with plasma membrane lipid raft-enriched detergent-resistant membranes (DRMs) of Huh-7 cells [43]. Our analysis of the localization of SR-BI revealed that SR-BI staining, which was largely cytoplasmic, frequently colocalized CD81 at the cell surface and this colocalization was not appreciably altered by PDZK1 knockdown. Similarly, we did not observed any marked effects of PDZK1 knockdown on the staining pattern of occludin.

Based on what is known of the kinetics of antibody-mediated inhibition of HCV entry [15], [44], [45] and the differences in the localization of the four major HCV entry factors in human liver and hepatoma cells that support HCV entry [39], [46], [47], it has been suggested that SR-BI and CD81 are involved in post-binding steps of HCV entry, after which lateral migration of virus/receptor complexes towards tight junctions occurs and interaction with CLDN1 and occludin takes place.

Tight junctions are likely to be largely inaccessible to HCV under normal physiological conditions. Indeed, recent studies involving the use of the colorectal adenocarcinoma cell line Caco-2 that develops simple columnar polarity and HepG2 hepatoma cells that develop complex hepatic polarity have demonstrated that the formation of genuine tight junctions between cells as they develop polarity results in restricted access to viral receptors and inhibition of HCV entry [39], [48]. Interestingly, however, disruption of the integrity of tight junctions of polarized HepG2-CD81 cells with inflammatory cytokines does not perturb cell polarity or enhance HCV entry, whereas phorbol ester-induced activation of protein kinase C (PKC) results in both disruption of junctional integrity and cellular polarity and enhancement of HCV entry [39]. Similarly cAMP-dependent activation of protein kinase A (PKA) has also been shown to be important to HCV entry, with inhibition of PKA activity causing redistribution of CLDN1 to intracellular sites, disruption of CLDN1 association with CD81 and inhibition of HCV entry [49]. Intriguingly PKA activation is also associated with phosphorylation of PDZK1 [33], suggesting that the effects of PKA activity on HCV entry may also coincide with PDZK1-dependent changes in the involvement of SR-BI in the entry process. This possibility warrants further investigation. The involvement of HCV itself in the disruption of tight junctions and enhancement of HCV entry is also gaining attention. For example, HCV infection has been associated with disruption of the localization of claudin-1 and occludin to sites of cell-cell contact [50] and modulation of CD81 homodimerization and CD81 heterodimerization with claudin-1 [51]. Furthermore, a recent study has revealed that vascular endothelial growth factor (VEGF), which is upregulated in HCV-infected hepatocytes, promotes disruption of hepatocyte polarity and tight junctions between cells, thereby increasing their susceptibility and the susceptibility of nearby cells to HCV infection [52]. Further studies are required to determine whether disruption of cellular polarity and HCV infection have any impact upon the subcellular localization of SR-BI and PDZK1.

In argument against a rate-limiting role for PDZK1 in HCV entry the degree of inhibition of viral entry observed in PDZK1-knockdown Huh-7 cells (30-80%) did not closely reflect the degree of knockdown of PDZK1 protein levels (>90%). Although it could be argued that residual amounts of PDZK1 protein are sufficient to partially compensate for the knockdown of the majority of endogenous PDZK1 expression, we could not detect surface-associated PDZK1 in streptavidin precipitates of biotinylated plasma membrane proteins prepared from these cells indicating that residual amounts of PDZK1 were not preferentially associated with SR-BI at the cell-surface. Previously it was shown that a truncated mutant of SR-BI (SR-BIdel509), that does interact with PDZK1, was not readily detectable at the plasma membrane of hepatocytes prepared from SR-BIdel509 transgenic mice, indicating that in polarized hepatocytes interaction with PDZK1 is required for the correct localization of SR-BI [27]. Somewhat paradoxically, hepatic overexpression of full-length SR-BI in PDZK1-knockout mice can restore wildtype levels of total and surface SR-BI protein and reverse the effects of PDZK1-knockout on HDL metabolism [30], suggesting that alternative and likely inefficient PDZK1-independent means for the effective enrichment of full-length SR-BI at the hepatocyte plasma membrane exist. Further studies involving the use of polarized hepatoma cells or primary hepatocytes in which PDZK1 expression has been ablated are required to accurately predict the relative importance of PDZK1 to HCV infection in the human liver *in vivo*. Given recent major advances in the identification of factors that determine human hepatotropism of HCV entry [18] and adaptation of HCVcc to murine CD81 [53], studies of HCV entry into primary hepatocytes isolated from PDZK1-KO mice and derivatives may be possible in the foreseeable future.

It has recently been reported that PDZK1-knockdown does not have a substantial effect on HCVpp infection or HDL-mediated enhancement of HCVpp infection [12]. Although the reasons for the differences between the findings of that study and ours are not obvious, it is possible that near-complete knockdown of endogenous PDZK1 is required to observe inhibition of HCV entry. It is also possible that, between Huh-7 cell derivatives, variations in the relative levels of each of the HCV entry factors influences the relative dependence on that protein for efficient HCV entry, such that high levels of CD81, for example, could result in decreased dependence on SR-BI for initial capture of cell-free virus. Nevertheless, we employed additional overexpression and dominant-negative strategies to further investigate the importance of SR-BI/PDZK1 interaction to HCV entry. These studies revealed that overexpression of full-length PDZK1 significantly increased HCVcc infection levels in Huh-7 cells and restored baseline HCVcc infection levels in Huh-7 cells displaying knockdown of endogenous PDZK1. In contrast the SR-BI-interacting domain (PDZ1) of PDZK1 alone was not sufficient to restore normal HCVcc infection levels in PDZK1 knockdown cells indicating that regions outside this domain are required. Relevant to this, all four PDZ domains of PDZK1 are required to restore wildtype SR-BI protein levels and function in lipoprotein metabolism in PDZK1 knockout mice [31]. The reasons that PDZ1 overexpression did not have an inhibitory impact on HCVcc infection, as might be expected given the dominant-negative effects of hepatic PDZ1 (amino acids 1–116) expression on SR-BI expression and function in transgenic mice [32], are not clear. However the additional nuclear localization of our PDZ1 construct (amino acids 1–130) that was not observed in PDZ1-transgenic mice indicates the presence of substantive differences between the constructs that were not anticipated. Nevertheless, expression of the PDZK1-interacting domain of SR-BI, as a GFP fusion protein, resulted in reduced susceptibility of Huh-7 cells to HCVcc infection and a cytoplasmic organellar localization of the fusion protein. We therefore propose that pharmaceutical mimicry of this PDZK1-interacting domain of SR-BI may represent a future target of anti-HCV therapy which is relatively liver-specific.

Interestingly, like SR-BI, occludin and claudin-1 also interact with PDZ domain-containing cytoplasmic adaptor molecules (ZO-1 and ZO-2, respectively) via their cytoplasmic carboxy-termini (reviewed in [54]). While knockdown of ZO-1 expression has been associated with a decreased susceptibility of Huh-7 cells to HCV entry [16], this effect was not observed in another similar study [46]. Similarly, although C-terminally truncated claudin-1 can still support HCV entry, this mutation was associated with an approximately 3-fold reduction in HCV entry [15], suggesting that the PDZ-interacting domain of claudin-1 contributes to its efficient involvement in HCV entry. Interactions of SR-BI, occludin and claudin-1 with their respective PDZ domain-containing adaptor molecules at the inner leaflet of the plasma membrane may be particularly important to their respective localizations and activities in polarized hepatocytes in vivo.

Taken together our results indicate that the interaction of PDZK1 with SR-BI contributes to the efficient infection of hepatoma cells by HCVcc. Since the identification of what may represent the complete set of essential HCV entry factors, it remains to be determined when and how each of these proteins participates in viral entry and the secondary factors that influence dynamic changes in the localization and activity of each of the HCV entry factors. Future studies of this nature will improve our understanding of HCV infection and tropism and may reveal new targets of antiviral therapy.

## Materials and Methods

### Cell culture

A Huh-7 cell line that is permissive to HCV infection and replication was kindly provided by Eric Gowans (Children's Health Research Institute, Adelaide). Huh-7 cells harbouring the genotype 1b NNeo/C-5B(RG) genomic HCV replicon [55] were kindly provided by Stanley Lemon (University of Texas Medical Branch, Galveston). The HepG2(N6) clone was kindly provided by David Anderson (Macfarlane Burnet Institute, Melbourne). 293T cells were obtained from the American Type Culture Collection. With the exception of HepG2(N6) cells and their derivatives which were culture as previously described [38], all cells were maintained in Dulbecco's Modified Eagle Medium (DMEM) supplemented with 2 mM L-glutamine, non-essential amino acids, penicillin (100 U/ml), streptomycin (100 µg/ml) and 10% foetal bovine serum (FBS). Where appropriate, selective antibiotics G418 (800 µg/ml), puromycin (3 µg/ml) and/or blasticidin (3 µg/ml) were added to culture media. All cells were cultured at 37°C in 5% CO_2_.

### Antibodies and conjugates

Rabbit polyclonal antibodies against PDZK1 (Zymed Laboratories), SR-BI (Novus Biologicals), C-myc (Santa Cruz) and FLAG (Sigma) were purchased. Mouse monoclonal antibodies against the following antigens were purchased: FLAG (M2; Sigma), β-actin (clone AC-15; Sigma), C-myc (clone 9E10; Roche Applied Science), phosphoserine (clone 7F12; Invitrogen), CD81 (clone JS81; Pharmingen), and occludin (clone OC-3F10; Zymed Laboratories). Biotinylated goat anti-GFP antibody was purchased from Rockland Immunochemicals. Human anti-SR-BI monoclonal antibody C-167 [56] was kindly provided by Alessandra Vitelli (Istituto di Ricerche di Biologia Molecolare P. Angeletti, Rome). Mouse monoclonal anti-NS5A antibody 9E10 was kindly provided by Charles Rice (The Rockefeller University, New York). Alexa Fluor-488 and -555 conjugated secondary antibodies were purchased from Invitrogen. HRP-conjugated anti-mouse IgG and anti-rabbit IgG antibodies and streptavidin-conjugated HRP were from Pierce.

### Plasmids and transfection

The following plasmids were generous gifts: pJFH-1 [7] (Takaji Wakita; National Institute of Infectious Diseases, Shinjuku-ku), pJc1/GFP and pJc1/RFP [36] (Ralf Bartenschlager; University of Heidelberg, Heidelberg), pER-RFP and pGolgi-RFP [57] (Erik Snapp; Albert Einstein College of Medicine, New York) and pLenti6/V5-D-TOPO-tdTomato (Yuka Harata-Lee; University of Adelaide, Adelaide). Plasmid pJc1/Myc was generated by in-frame replacement (*Xba*I/*Rsr*II) of the GFP coding region of pJc1/GFP with an oligonucleotide adaptor duplex encoding the C-myc epitope (EQKLISEEDL). PDZK1 cDNA (NM_002614) expression constructs with C-terminal FLAG (DYKDDDDK) epitope tags, PDZK1-FLAG and PDZ1-FLAG (encoding aa 1–130), were cloned into pcDNA3 (*Hind*III/*Xba*I) (Invitrogen) and pLenti6/V5-D-TOPO (*Bam*HI/*Xho*I) (Invitrogen). Where indicated mutations encoding the amino acid substitutions S505A or S505D (referred to as S509A and S509D for consistency with the original description of Ser-509 phosphorylation in rat PDZK1 [33]), and the silent mutation S^tca^261S^agc^ were introduced by QuickChange site-directed mutagenesis (Stratagene). Full-length and C-terminally truncated (Δ509) SR-BI cDNA (NM_005505) expression constructs, with or without N-terminal Myc or FLAG epitope tags, were cloned into pcDNA6/V5-HisB (*Hind*III/*Xba*I) (Invitrogen). EGFP-WT-ctt and EGFP-Δ509-ctt expression plasmids were generated by cloning the cDNA region encoding the cytoplasmic C-terminus of SR-BI (aa 479–509) with a Myc epitope tag at the N-terminus into pcDNA3 (*Xho*I/*Xba*I). The EGFP coding sequence, with the stop codon removed, was then cloned in-frame into upstream restriction sites (*Eco*RI/*Xho*I). To generate pcDNA3-EGFP the EGFP coding sequence, including the stop codon, was cloned into pcDNA3 (*Eco*RI/*Xho*I). Plasmid pcDNA3-CD81myc was generated by cloning CD81 cDNA (NM 004356), with a C-terminal C-myc epitope tag, into pcDNA3 (*Hind*III/*Bam*HI). All constructs were confirmed by automated DNA sequencing. Exact cloning details are available upon request. To generate HepG2(N6) cell lines expressing CD81, HepG2(N6) cells were transfected with pcDNA3-CD81myc using FuGene6 (Roche) as per manufacturer's instructions and, 48 h post-transfection, G418 (800 µg/ml) selection was applied. G418-resistant clones were later individually expanded and screened for CD81 expression by flow cytometry (not shown). One clone displaying uniform, high-level expression of CD81 was chosen for further analysis. Huh-7 cells stably expressing EGFP, EGFP-WT-ctt or EGFP-Δ509-ctt were generated by transfection with the corresponding pcDNA3 expression plasmid, selection with G418 and enrichment of GFP-positive cells using a FACSAria flow cytometer (BD Biosciences).

### Virus generation and infections

Cell culture propagated HCV particles (HCVcc; JFH-1, Jc1/GFP, Jc1/RFP and Jc1/Myc) were generated as described previously [8]. Briefly, *in vitro* transcribed HCV RNA was generated from *Xba*I-linearized plasmid pJFH-1 or *Mlu*I-linearized plasmids pJc1/GFP, pJc1/RFP or pJc1/Myc using a MEGAscript T7 kit (Ambion). Huh-7 cells were electroporated with 10 µg of RNA and at 5 d post-transfection (3 d post-transfection for Jc1/GFP and Jc1/RFP preparations) virus containing supernatants were collected, filtered (0.45 µm) and used undiluted to infect naïve cells for 3 hours. Cell monolayers were then washed twice with PBS and returned to culture for 48–72 h, as indicated, prior to analysis of HCV infection. HCVcc infectious titres (focus forming units [FFU]/ml) were determined as described previously [8].

Pseudoviruses encoding Luciferase were generated by co-transfection of 293T cells with the lentiviral packaging plasmids psPAX2 (Addgene plasmid 12260), pRSV-Rev (Addgene plasmid 12253), pLenti6/V5-D-TOPO-Luciferase and either VSV-G envelope expression plasmid pMD2.G (Addgene plasmid 12259) for generation of VSVpp, the expression plasmid pE1E2H77c [4] for generation of HCVpp or empty plasmid pcDNA3 for generation of Env-pp. Supernatants were harvested at 48 and 72 h post-transfection, pooled and filtered (0.45 µm). Virus-containing cell culture supernatants were then diluted in normal culture media (1∶500 for VSVpp, 1∶2 for HCVpp and Env-pp) and incubated with target cells, seeded at 2×10^4^ cells/cm^2^ the day before (unless otherwise specified), for 7 h before washing with PBS and return to culture. Pseudoparticle infections were performed in the presence of 4 µg/ml polybrene. At 72 h post-infection luciferase activity was measured using a Luciferase Assay System (Promega) and a GloMax 96 microplate luminometer (Promega). Specific HCVpp and VSVpp infectivity levels were determined by subtraction of the luciferase signals associated with the use of non-enveloped pseudoparticles (Env-pp).

Lentiviral pLKO.1 shRNA constructs specific for human PDZK1 were purchased from Open Biosystems (target set for NM_002614). PDZK1-specific shRNA clones (PDZK1 shRNA #4, TRCN0000059671; PDZK1 shRNA#5, TRCN0000059672) and a control non-target shRNA clone (Sigma; SHC002) were packaged into lentiviral vectors by co-transfection of 293T cells along with psPAX2 and pMD2.G. Lentiviral overexpression vectors were generated by co-transfection of 293T cells with psPAX2, pMD2.G, pRSV-Rev and either pLenti6/V5-D-TOPO-PDZK1-FLAG or pLenti6/V5-D-TOPO-PDZ1-FLAG. Lentiviral supernatants were collected at 48 and 72 h post-transfection, pooled, filtered (0.45 µm) and stored at −80°C. Target cells were infected for 4 h with lentiviral supernatants diluted 1∶5 in normal culture medium containing 8 µg/ml polybrene (Sigma). Antibiotic selection with puromycin ([3 µg/ml] for pLKO.1 constructs) or blasticidin ([3 µg/ml] for pLenti6/V5-D-TOPO constructs) was applied 48 h later to generate antibiotic-resistant polyclonal cell lines.

### Laser scanning confocal microscopy

Huh-7 cells were grown on 0.2% gelatin-coated coverslips overnight, transfected where applicable and fixed the following day in ice-cold acetone:methanol (FLAG, Myc, NS5A) or ice-cold 5% buffered formalin (GFP/RFP epifluorescence, SR-BI, CD81, occludin, LAMP1). Where appropriate, cells were permeabilized with 0.05% saponin in PBS, prior to blocking in 5% BSA in PBS and incubation with primary antibody diluted in 1% BSA in PBS for 1 h at room temperature. After washing twice with PBS, Alexa Fluor-488 and/or Alexa Fluor-555 conjugated secondary antibodies were applied for 1 h at 4°C. Cell monolayers were then washed twice with PBS and coverslips were mounted using ProLong Gold antifade reagent (Invitrogen). Where combinations of antibodies were used, control experiments using isotype-matched irrelevant antibodies or secondary antibody alone (SR-BI surface labelling) were performed to ensure minimal non-specific binding of antibodies. Cells were viewed using an Olympus IX70 inverted microscope linked to a Bio-Rad Radiance 2100 confocal microscope. Samples were visualized using a ×60/1.4NA water immersion lens (2–3× zoom) and images were acquired sequentially for each fluorophore and processed using Adobe Photoshop 6.0 software (Adobe Systems Inc).

### HCV RNA quantitation

Extraction of total cellular RNA, first-strand cDNA synthesis and real-time RT-PCR was performed as described [58].

### Immunoprecipitation

Immunoprecipitation of FLAG-tagged proteins was performed as follows. Near-confluent cells in 35 mm dishes were washed with PBS before lysis in 0.5 ml of NP-40 lysis buffer (1% NP-40, 150 mM NaCl, 50 mM Tris [pH 8.0]) containing protease inhibitors (Sigma) on ice for 30 min. Samples were then passed through a 26 gauge needle 10 times and cleared of nuclear debris by centrifugation (10,000×g, 10 min). All incubations were performed at 4°C with end-over-end rotation. Lysates were pre-cleared by incubation with 20 µl of protein A/G PLUS agarose (Santa Cruz Biotechnology) for 1 h. Samples were then centrifuged (1000×g, 5 min, 4°C) and supernatants were collected and incubated with 1 µg of anti-FLAG mAb overnight. 20 µl of protein A/G PLUS agarose was then added to each sample prior to incubation for 1 h. Beads were then pelleted by centrifugation (1000×g, 5 min, 4°C) and washed 5 times with 1 ml of NP-40 lysis buffer before resuspension in 40 µl of SDS-PAGE loading buffer and Western analysis.

### Cell surface biotinylation and streptavidin precipitation

Biotinylation of plasma membrane proteins with membrane impermeable sulfo-NHS-biotin (Pierce) and streptavidin-precipitation of biotinylated proteins was performed as described previously [59].

### Western blotting

Cells were lysed in RIPA buffer (150 mM NaCl, 1% NP-40, 0.5% sodium deoxycholate, 0.1% SDS, 50 mM Tris, pH 8.0) containing protease inhibitors (Sigma) on ice for 30 min, homogenized and cleared by centrifugation (10,000×g, 10 min). For each sample approximately 30 µg of protein was separated by 12% SDS-PAGE and transferred to HyBond-ECL nitrocellulose membrane (Amersham Biosciences) for immunoblotting using anti-FLAG, anti-Myc, anti-GFP, anti-PDZK1, anti-SR-BI or anti-β actin. HRP-conjugated secondary antibodies or HRP-conjugated streptavidin were detected by chemiluminescence using SuperSignal West Femto (Pierce). Where indicated densitometric analysis of bands was performed using NIH Image software.

### Flow cytometry

Cells were harvested by trypsinization, washed (PBS/1%FBS) and incubated at 4°C for 1 h with appropriately diluted anti-CD81, anti-SR-BI (C-167), irrelevant isotype-matched control MAb (CD81 labelling) or no primary antibody (SR-BI labelling). Following washing, cells were incubated for 1 h at 4°C with Alexa Fluor-555 conjugated goat anti-mouse IgG or Alexa Fluor-555 conjugated goat anti-human IgG. Cells were then washed, fixed (1% formalin in PBS containing 111 mM D-glucose and 10 mM NaN_3_) and analysed using a FACS-Canto flow cytometer (BD Biosciences).

### Statistical analysis

Data are expressed as means + the standard error of the mean (SEM). Statistical analysis was performed by Student's *t* test, with *P*<0.05 considered to be statistically significant.

### Accession numbers

PDZK1, NM_002614; SR-BI, NM_005505; CD81, NM_004356; Claudin-1, NM_021101; Occludin, NM_002538; HCV (JFH-1), AB047639; HCV (HC-J6), HPCPOLP; HCV (H77), NC_004102.

## Supporting Information

Figure S1 Dimerization of SR-BI is independent of interaction with PDZK1. (A) 293T cells were co-transfected with the indicated epitope-tagged SR-BI expression vectors prior to immunoprecipitation of FLAG-tagged proteins and immunoblot detection of co-immunoprecipitated Myc-tagged proteins (lower panel). Mutation of the PDZK1-interacting domain of SR-BI (mycSR-BIΔ509) did not abrogate co-immunoprecipitation with FLAG-SR-BI or FLAG-SR-BIΔ509. (B) Huh-7 cells were co-transfected with the indicated epitope-tagged SR-BI or PDZK1 expression vectors prior to immunoprecipitation of FLAG-tagged proteins and immunoblot detection of co-immunoprecipitated Myc-tagged proteins (lower panel). These results suggest that dimerization of SR-BI in Huh-7 cells does not involve PDZK1 interaction and indicate that SR-BI/PDZK1 interaction occurs in Huh-7 cells as it does in 293T cells. (C) Confocal analysis of the localization of mycSR-BIΔ509 with respect to FLAG-SR-BI (middle panels) and PDZK1-FLAG (lower panels) revealed no discernable impact of C-terminal truncation of SR-BI on its localization.
(0.96 MB TIF)

Figure S2 Colocalization of SR-BI with CD81 is unaffected by PDZK1 knockdown. (A) Huh-7 cells expressing a non-target control shRNA or PDZK1 shRNA #5 were grown on coverslips, fixed and surface labelled with antibodies directed against SR-BI (mAb C-167; green) and CD81 (red). Merged images revealed extensive colocalization. (B) Alternatively these cells were fixed, labelled with anti-SR-BI (green), fixed again and permeabilized prior to indirect immunofluorescent labelling of occludin (red). Merged images revealed minimal overlap in the localization of these proteins. For these experiments z-sections (0.5 µm steps) were collected and representative images for a single optical slice are shown. For all combinations of antibodies parallel samples were labelled with secondary antibody only (for SR-BI labelling) or irrelevant isotype-matched control antibodies to confirm specificity of labelling (not shown).
(4.38 MB TIF)

Figure S3 Cell surface and intracellular localizations of SR-BI in Huh-7 cells expressing control or PDZK1-specific shRNAs. Huh-7 cells expressing a non-target control shRNA, PDZK1 shRNA #4 or PDZK1 shRNA #5 were grown on coverslips, fixed and surface labelled with anti-SR-BI (mAb C-167; green). Cells were then fixed again, permeabilized and labelled with a rabbit polyclonal antibody directed against the C-terminus of SR-BI (red). Merged images indicated that the majority of surface SR-BI/II labelling can be attributed to SR-BI. For these experiments z-sections (0.5 µm steps) were collected and representative images for a single optical slice are shown. Parallel samples were labelled with secondary antibody only (for SR-BI/II labelling) or irrelevant rabbit antisera (for SR-BI labelling) to confirm specificity of labelling (not shown).
(2.04 MB TIF)

Figure S4 HCV replication is unaffected by PDZK1 knockdown. (A) Huh-7 cells that harboured the genomic HCV replicon NNeo/C-5B(RG) were stably transduced with the indicated lentiviral shRNA vector prior to Western analysis of total SR-BI (∼85 kDa) and PDZK1 (∼70 kDa) protein levels. β-actin (∼42 kDa) served as a loading control. (B) Total RNA was extracted from these cells for real-time RT-PCR analysis of HCV RNA levels, normalized to RPLPO mRNA. Data are means + SEM (n = 4). (C) Indirect immunofluorescence detection of HCV antigens (using pooled antisera from HCV-infected individuals) in Huh-7 cells harbouring the NNeo/C-5B(RG) replicon and expressing the indicated shRNA constructs.
(0.70 MB TIF)

Figure S5 (A) Huh-7 cells stably expressing EGFP, EGFP-WT-ctt or EGFP-Δ509-ctt were surface biotinylated prior to detergent lysis and streptavidin precipitation of plasma membrane proteins. Western analysis of streptavidin precipitates revealed no appreciable impact of EGFP-WT-ctt expression upon surface levels of SR-BI. (B) Confocal analysis of EGFP-Δ509-ctt localization in Huh-7 cells. Huh-7 cells stably expressing EGFP-Δ509-ctt were grown on coverslips, transfected with the indicated expression plasmid (ER-RFP, Golgi-RFP, or RFP), fixed and processed for laser scanning confocal microscopy. Alternatively these cells were fixed and LAMP1 was labelled by indirect immunofluorescence (bottom panels). EGFP-Δ509-ctt was indistinguishable from unmodified EGFP in localization. Where appropriate parallel samples of non-transfected cells and/or isotype control labelled cells (for anti-LAMP1 labelling) were visualized to confirm specificity of labelling (not shown).
(1.31 MB TIF)
