# Supplementary figures and images for: The SR-BI Partner PDZK1 Facilitates Hepatitis C Virus Entry
Source: PLoS Pathog. 2010 Oct 7;6(10):e1001130. doi: 10.1371/journal.ppat.1001130 (PMC2951368; doi:10.1371/journal.ppat.1001130)

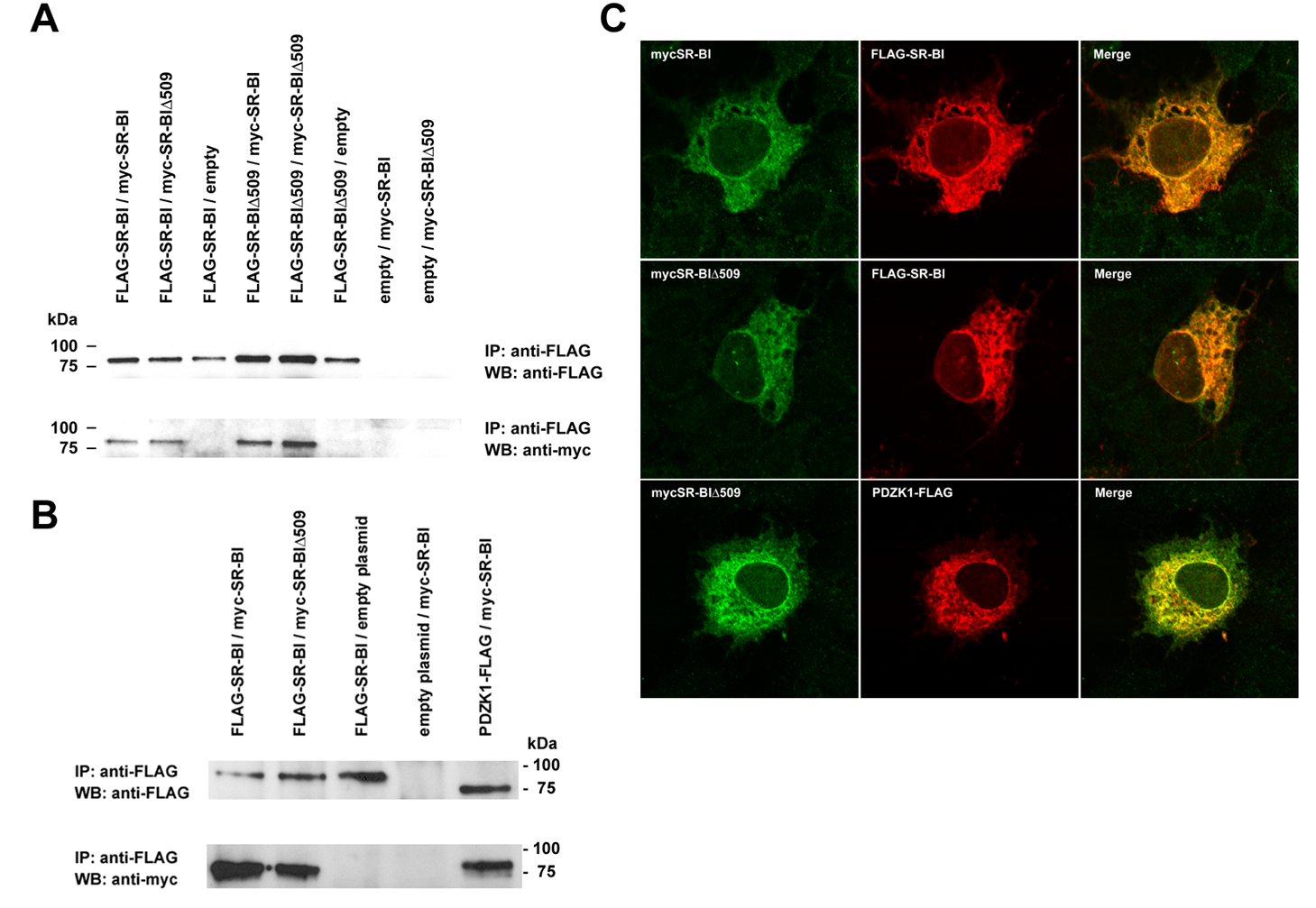

Supplement: Figure S1 — Dimerization of SR-BI is independent of interaction with PDZK1. (A) 293T cells were co-transfected with the indicated epitope-tagged SR-BI expression vectors prior to immunoprecipitation of FLAG-tagged proteins and immunoblot detection of co-immunoprecipitated Myc-tagged proteins (lower panel). Mutation of the PDZK1-interacting domain of SR-BI (mycSR-BIΔ509) did not abrogate co-immunoprecipitation with FLAG-SR-BI or FLAG-SR-BIΔ509. (B) Huh-7 cells were co-transfected with the indicated epitope-tagged SR-BI or PDZK1 expression vectors prior to immunoprecipitation of FLAG-tagged proteins and immunoblot detection of co-immunoprecipitated Myc-tagged proteins (lower panel). These results suggest that dimerization of SR-BI in Huh-7 cells does not involve PDZK1 interaction and indicate that SR-BI/PDZK1 interaction occurs in Huh-7 cells as it does in 293T cells. (C) Confocal analysis of the localization of mycSR-BIΔ509 with respect to FLAG-SR-BI (middle panels) and PDZK1-FLAG (lower panels) revealed no discernable impact of C-terminal truncation of SR-BI on its localization. (0.96 MB TIF) [file ppat.1001130.s001.tif]

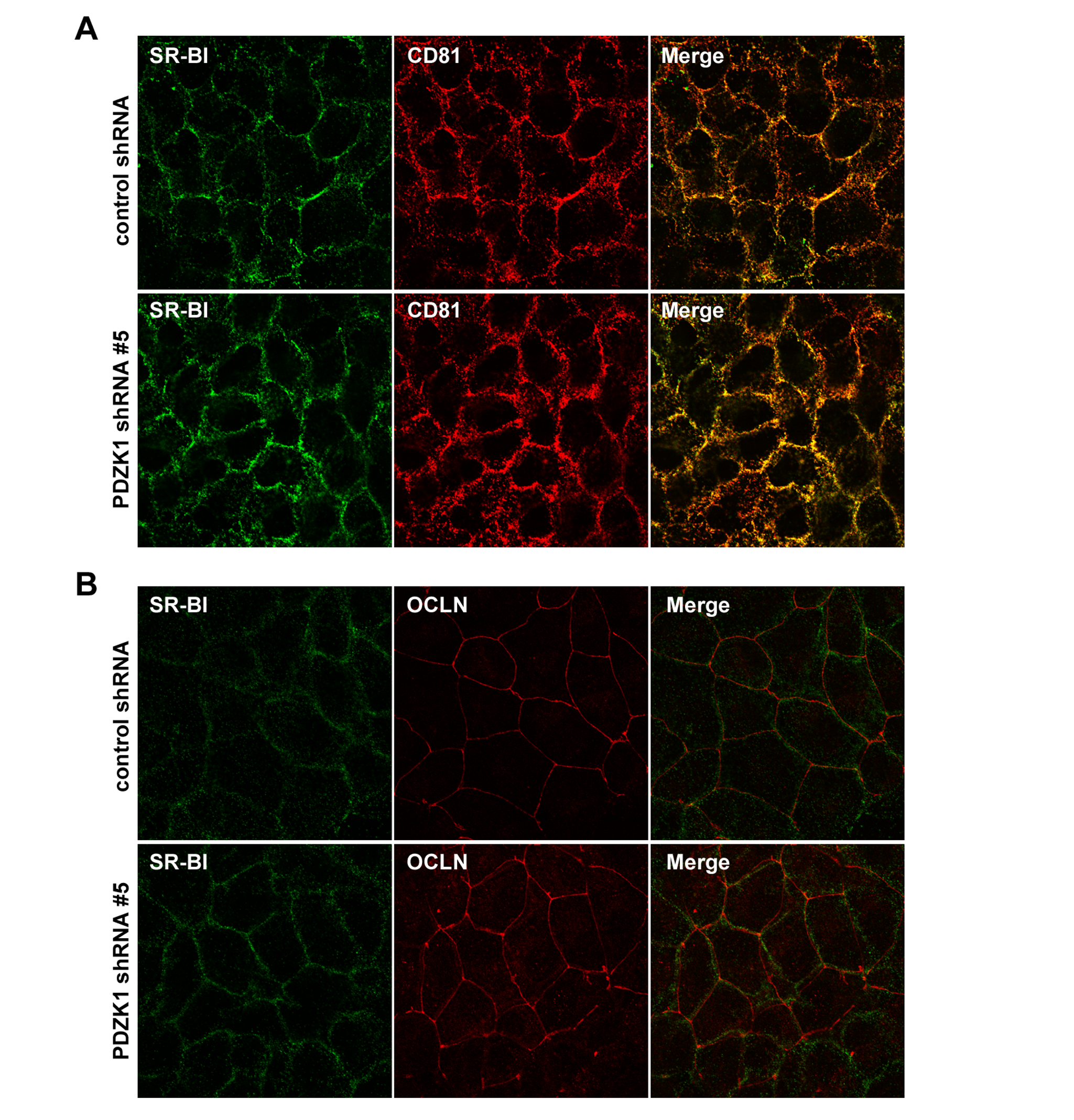

Supplement: Figure S2 — Colocalization of SR-BI with CD81 is unaffected by PDZK1 knockdown. (A) Huh-7 cells expressing a non-target control shRNA or PDZK1 shRNA #5 were grown on coverslips, fixed and surface labelled with antibodies directed against SR-BI (mAb C-167; green) and CD81 (red). Merged images revealed extensive colocalization. (B) Alternatively these cells were fixed, labelled with anti-SR-BI (green), fixed again and permeabilized prior to indirect immunofluorescent labelling of occludin (red). Merged images revealed minimal overlap in the localization of these proteins. For these experiments z-sections (0.5 µm steps) were collected and representative images for a single optical slice are shown. For all combinations of antibodies parallel samples were labelled with secondary antibody only (for SR-BI labelling) or irrelevant isotype-matched control antibodies to confirm specificity of labelling (not shown). (4.38 MB TIF) [file ppat.1001130.s002.tif]

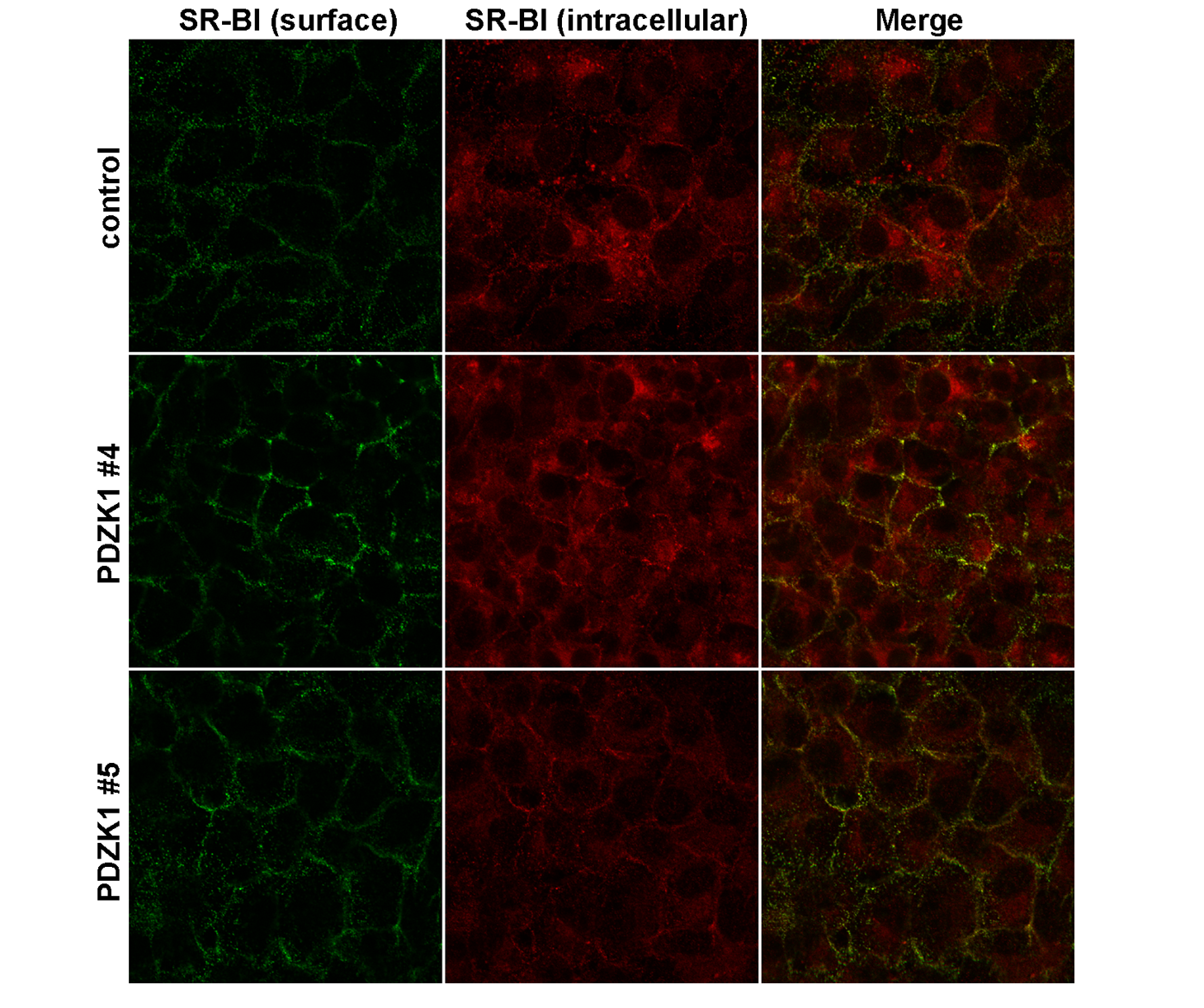

Supplement: Figure S3 — Cell surface and intracellular localizations of SR-BI in Huh-7 cells expressing control or PDZK1-specific shRNAs. Huh-7 cells expressing a non-target control shRNA, PDZK1 shRNA #4 or PDZK1 shRNA #5 were grown on coverslips, fixed and surface labelled with anti-SR-BI (mAb C-167; green). Cells were then fixed again, permeabilized and labelled with a rabbit polyclonal antibody directed against the C-terminus of SR-BI (red). Merged images indicated that the majority of surface SR-BI/II labelling can be attributed to SR-BI. For these experiments z-sections (0.5 µm steps) were collected and representative images for a single optical slice are shown. Parallel samples were labelled with secondary antibody only (for SR-BI/II labelling) or irrelevant rabbit antisera (for SR-BI labelling) to confirm specificity of labelling (not shown). (2.04 MB TIF) [file ppat.1001130.s003.tif]

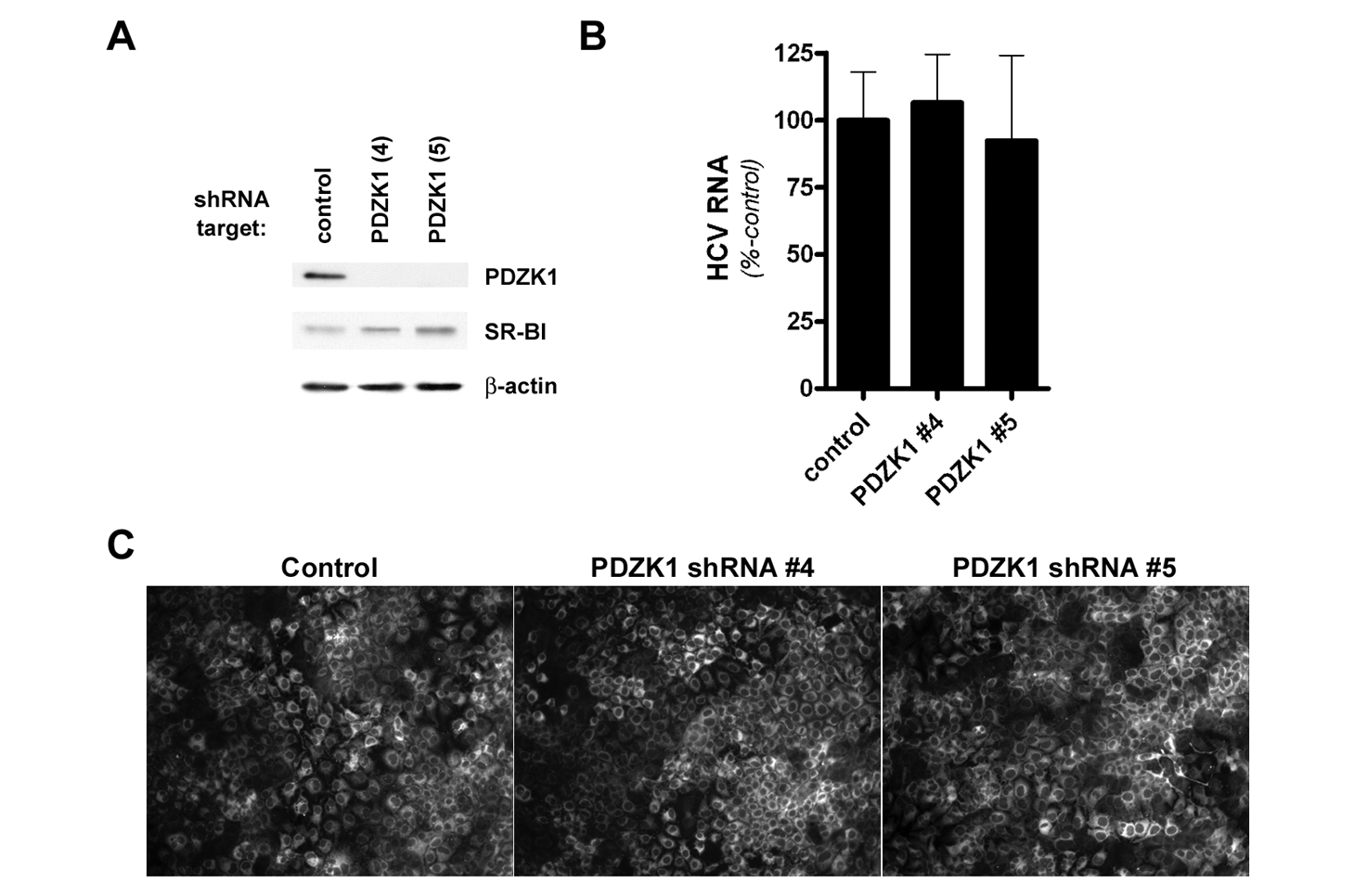

Supplement: Figure S4 — HCV replication is unaffected by PDZK1 knockdown. (A) Huh-7 cells that harboured the genomic HCV replicon NNeo/C-5B(RG) were stably transduced with the indicated lentiviral shRNA vector prior to Western analysis of total SR-BI (∼85 kDa) and PDZK1 (∼70 kDa) protein levels. β-actin (∼42 kDa) served as a loading control. (B) Total RNA was extracted from these cells for real-time RT-PCR analysis of HCV RNA levels, normalized to RPLPO mRNA. Data are means + SEM (n = 4). (C) Indirect immunofluorescence detection of HCV antigens (using pooled antisera from HCV-infected individuals) in Huh-7 cells harbouring the NNeo/C-5B(RG) replicon and expressing the indicated shRNA constructs. (0.70 MB TIF) [file ppat.1001130.s004.tif]

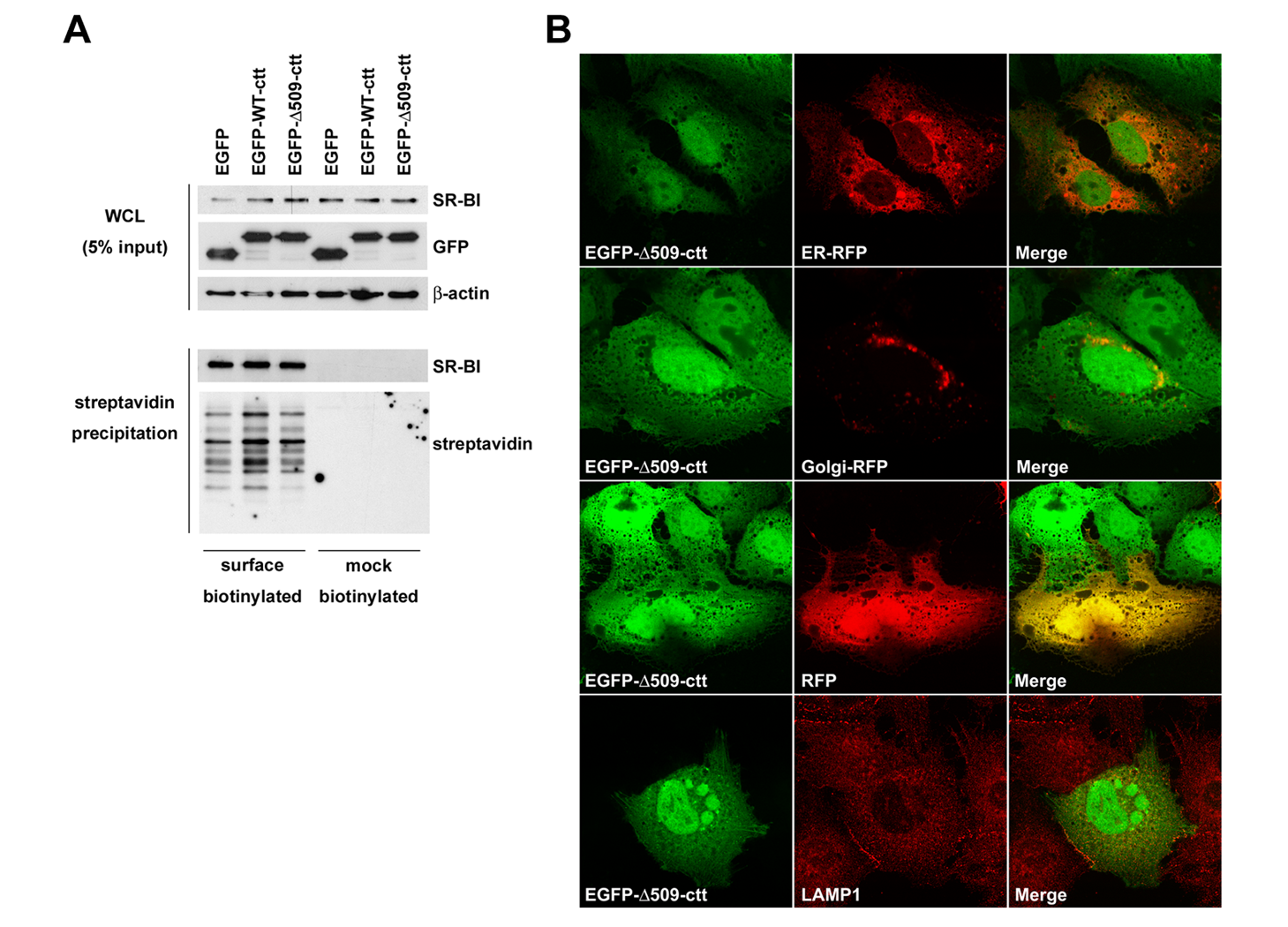

Supplement: Figure S5 — (A) Huh-7 cells stably expressing EGFP, EGFP-WT-ctt or EGFP-Δ509-ctt were surface biotinylated prior to detergent lysis and streptavidin precipitation of plasma membrane proteins. Western analysis of streptavidin precipitates revealed no appreciable impact of EGFP-WT-ctt expression upon surface levels of SR-BI. (B) Confocal analysis of EGFP-Δ509-ctt localization in Huh-7 cells. Huh-7 cells stably expressing EGFP-Δ509-ctt were grown on coverslips, transfected with the indicated expression plasmid (ER-RFP, Golgi-RFP, or RFP), fixed and processed for laser scanning confocal microscopy. Alternatively these cells were fixed and LAMP1 was labelled by indirect immunofluorescence (bottom panels). EGFP-Δ509-ctt was indistinguishable from unmodified EGFP in localization. Where appropriate parallel samples of non-transfected cells and/or isotype control labelled cells (for anti-LAMP1 labelling) were visualized to confirm specificity of labelling (not shown). (1.31 MB TIF) [file ppat.1001130.s005.tif]
